# Clinical Decision Making, Red-Flag Recognition, and Scope-of-Practice Perspectives for Direct Access Physical Therapy in Saudi Arabia

**DOI:** 10.3390/healthcare14193347

**Published:** 2026-10-08

**Authors:** Msaad Alzhrani

**Affiliations:** Department of Physical Therapy and Rehabilitation, College of Applied Medical Sciences, Majmaah University, Al Majma’ah 11952, Saudi Arabia; m.alzhrani@mu.edu.sa

**Keywords:** direct access, physical therapy, patient safety, clinical decision-making, red flags, scope of practice, Saudi Arabia

## Abstract

**Background/Objectives:** This survey examined referral decisions, the recognition and screening of warning features (red flags) of serious disease, regulatory interpretations, and barriers to direct-access physical therapy in Saudi Arabia. **Methods:** An English-language cross-sectional convenience survey (May–July 2026) used an investigator-developed questionnaire with 12 clinical vignettes (written patient cases) and 20 warning-feature items paired with screening questions. The primary outcome was choosing medical referral before physical therapy in all four urgent/critical vignettes: suspected gastrointestinal bleeding, cauda equina syndrome, abdominal aortic aneurysm, and spinal infection. This definition was finalised after data collection. **Results:** Of 131 Saudi physical therapists, 77 (58.8%; 95% confidence interval [CI] 50.2–66.8) chose referral before treatment in all four vignettes. Another 36 included referral in every vignette but would also start treatment while arranging referral in at least one; 18 chose treatment without referral at least once. In an exploratory six-predictor analysis, postgraduate academic qualifications were associated with higher odds of meeting the primary outcome (adjusted odds ratio 4.14, 95% CI 1.63–10.51; Holm-adjusted *p* = 0.017) and older age with lower odds (0.92/year, 95% CI 0.86–0.98; Holm-adjusted *p* = 0.047). Across 17 core warning features, 74.0% of responses identified a red flag; 49.1% of screening responses indicated checking often or always. Regulatory views differed: 45.8% believed direct access was permitted in limited settings. The highest-rated barriers concerned policy, law, reimbursement, and physician support. **Conclusions:** These findings identify priorities for evaluating training, clarifying regulations, and coordinating referral pathways. Vignettes and self-reported practices cannot establish safety in clinical practice, show that qualifications improve decisions, or determine workforce readiness.

## 1. Introduction

Direct access allows patients to consult a physical therapist without prior referral. Evidence suggests that it may improve timeliness and reduce healthcare use without compromising outcomes, although effects depend on regulatory, organisational, educational, and referral arrangements [1,2].

Safe first-contact practice requires appropriate management and referral decisions, a recognition of possible serious pathology, and clear escalation pathways [3,4]. Saudi and Gulf studies have examined red-flag knowledge, attitudes, and implementation barriers, generally as separate domains [5,6,7].

### 1.1. Direct Access, Outcomes, and Governance

Direct access varies with legislation, reimbursement, sector, institutional policy, and credentialing. A global survey reported direct access in 58% of responding jurisdictions, which was more often in private settings [1]. Potential benefits therefore depend on defined professional authority, access to medical assessment, documentation, and safety monitoring [2].

### 1.2. Clinical Decision Making and Red-Flag Screening

Clinical vignettes standardise information to examine management decisions. Jette et al. reported correct decisions for approximately 87% of musculoskeletal, 88% of non-critical medical, and 79% of critical medical vignettes [3], but their validation evidence cannot be transferred to newly developed vignettes. Serious-pathology screening requires contextual reasoning because most individual red flags have limited diagnostic value in isolation [4]. Saudi research found favourable awareness but lower reported use [5]. Cancer history, neurological changes, infection risk, and other warning features must be interpreted with the presentation and consequences of delayed referral [4,8,9]. Vignette development also requires explicit management standards and transparent review [10]. Recognition, reported screening, confidence, and management decisions are consequently distinct constructs.

### 1.3. Saudi and Gulf Context, Evidence Gap, and Objectives

Direct access is distinct from extended privileges such as prescribing, injections, or ordering investigations. Saudi and Gulf evidence identifies legal, reimbursement, educational, and organisational constraints and uncertainty about permitted practice [6,7,11]. International competency frameworks extend beyond clinical knowledge to communication, collaboration, and professional practice [12]. Saudi licensing provisions and government-hospital guidance describe different service and referral arrangements [13,14,15], but they do not establish an authoritative nationwide self-referral standard. Partial permission in limited settings is therefore treated here as the investigator’s interpretation.

This study addressed the absence of a concurrent assessment of clinical decisions, warning-feature recognition and screening, confidence, regulation, scope, and implementation perspectives in one Saudi cohort [5,6,7,11]. An investigator-developed survey separated keyed responses from self-reported behaviour and attitudes. Saudi nationals were the primary policy-relevant cohort because the principal question concerned the national workforce; eligible non-Saudi therapists formed a secondary exploratory cohort. The primary outcome for this report was referral before intervention in all four urgent/critical vignettes. No directional hypotheses or overall ready/not-ready threshold were specified; association analyses were two-sided.

## 2. Materials and Methods

### 2.1. Study Design and Setting

This multi-regional, cross-sectional survey used an open English-language Google Forms questionnaire (Google LLC, Mountain View, CA, USA), 11 May–25 July 2026. Recruitment covered Saudi Arabia without implying national representativeness. STROBE and CHERRIES reporting checklists are supplied in Supplementary Files S6 and S7 [16,17].

### 2.2. Participants, Recruitment, Consent, and Sample Size

Eligible respondents self-reported current Saudi Commission for Health Specialties (SCFHS) registration, age ≥ 18 years, clinical physical therapy practice in Saudi Arabia, and ≥1 year of post-licensure experience. Interns, students, trainees, and people working exclusively in non-clinical academic or administrative roles were ineligible. Eligibility was not independently verified. Nationality was not an eligibility restriction: Saudi nationals formed the primary cohort and eligible non-Saudi therapists the secondary exploratory cohort.

Professional associations, institutional contacts, and professional networks distributed the link across sectors and the 13 administrative regions. Recruitment was voluntary convenience sampling. No technical one-response restriction or unique participant identifiers were used; duplicate participation could not be established. Dissemination and invitation-viewer counts were unrecorded, preventing the calculation of a conventional response rate. Required scored items, model covariates, and certification responses were complete among eligible participants; the open-text question was optional. Technical administration details are in Supplementary File S7.

Mandatory consent and professional-eligibility gates preceded study questions; a negative response ended the form. A pragmatic recruitment benchmark of 385 used a single-proportion calculation with 95% confidence, *p* = 0.50, and 5% precision [18] (File S4, Section S20). This benchmark was not reached and did not confer population representativeness. Reasons for non-participation were unknown.

### 2.3. Questionnaire and Administration

The author developed the questionnaire using clinical-vignette research [3,10], serious-pathology frameworks [4,5,8,9], direct-access and competency literature [1,6,7,11,12], and Saudi regulatory sources [13,14,15]. These informed content and structure; the questionnaire was not an unchanged validated instrument.

No independent prelaunch expert review, consensus procedure, or validation was undertaken; no separate pilot or technical usability pretest is documented. Final scoring specifications differed from earlier versions and were not established as precollection decisions. During revision, a targeted, non-systematic guideline-based appraisal examined 12 vignettes and 20 warning features with submitted keys and descriptive results available. It documents clinical support, exact-key uncertainty, and alternatives, but it is not independent expert validation or a contemporaneous development rationale (File S10).

The questionnaire comprised demographic/professional items, 12 clinical vignettes, and 20 warning-feature items grouped into seven domains (File S4, Table S3) with matched screening-frequency questions, confidence, regulatory and scope perspectives, stakeholder support, educational preparation, barriers, and one optional open-text question. The estimated completion time was 15–20 min. File S5 reproduces the administered questionnaire; File S4 summarises item grouping and scoring.

English was selected because professional familiarity was expected from physical therapy education and practice; proficiency was not assessed. The introductory message asked respondents to use workplace rules and experience; it is not reproduced in File S5. Vignette instructions also specified using only the information provided. An unrestricted direct-access model was therefore not uniformly stipulated, and workplace rules may have influenced responses.

Vignettes comprised six musculoskeletal, two medically concerning/non-critical, and four urgent/critical presentations. Response options were treat without referral (Option 1), treat with concurrent consultation/referral (Option 2), and refer before intervention (Option 3). This structure was informed by Jette et al. [3], but the vignettes were investigator-developed. Seventeen warning features (RF3–RF19) were keyed Yes; three contextual items (RF1, RF2, RF20) were keyed No under the investigator’s interpretation that they alone were insufficient. The latter interpretation was narrower than the question stem. Regulatory responses were reported in four categories; a secondary binary summary indicated agreement with the investigator’s limited-settings interpretation rather than verified legal knowledge.

The awareness stem asked whether each feature was a “RED FLAG warranting further investigation or referral” without separating suspicion, further assessment, and independent referral indications. Negatively keyed responses cannot establish clinical overcalling. All 17 core items were positively keyed, potentially favouring a general Yes-response tendency. Separate Yes, No, and Not sure counts are reported in File S4, Table S18b. Screening concerned initial patient assessment and specified no recall period.

The open-text question concerned training, support, or policy changes enabling direct access. Eight themes were defined before reading comments; multiple labels were permitted. Blank, punctuation-only, and explicit no-comment entries were excluded. During revision, the author reviewed all 32 substantive comments against their labels, corrected coding, and clarified two brief comments with AI assistance. Keyword matches were candidate flags; final assignments were encoded in R version 4.6.1. No independent second coder or translation check was used. File S8 documents coding rules, translations, ambiguous responses, and corrections; frequencies describe mentions rather than measured importance.

### 2.4. Outcomes and Scoring

The binary primary outcome required Option 3 for every urgent/critical vignette (V9–V12): suspected gastrointestinal bleeding, cauda equina syndrome, abdominal aortic aneurysm, and spinal infection. Secondary clinical measures were strict-key agreement (the submitted “strict-optimal accuracy” score, 0–12), study-defined graded scoring (0–24), referral-first vignette count (0–4), its complementary count, and Jette-compatible scoring (0–12). Strict scoring awarded one point for the preferred response(s); graded scoring awarded 2 for the preferred response, 1 for a specified alternative where applicable, and 0 otherwise. These weights do not classify clinical safety. Jette-compatible rules credited Options 1/2 for musculoskeletal, 2/3 for medically concerning, and 3 for urgent/critical vignettes. These keys and weights are study-defined; the submitted keys remain the baseline (File S4, Tables S2–S4).

Not meeting the primary criterion includes concurrent referral and does not establish unsafe care or a clinical error. The post hoc rationale distinguishes urgent same-day acute medical assessment before therapy for V9/V12, with immediate escalation if unstable, from immediate emergency pathways for V10/V11 [19,20,21,22]. These are suspected presentations of differing acuity. Referral destinations and guideline provisions are detailed in File S4, Table S16c, and File S10.

The post hoc appraisal supports medical assessment for V7’s progressive sensory symptoms and missed diabetes follow-up but does not establish that concurrent care is uniquely preferable to referral first [23]. Palpitations in V8 warrant assessment, including an ECG, but the abbreviated vignette does not establish appropriate treatment timing or a uniquely correct choice between Options 2 and 3 [24]. Its submitted key accepts both; referral-first-only scoring was therefore a stricter exploratory scenario rather than a guideline-mandated correction. For RF20, low-back-pain guidance supports reassessment after approximately one month without improvement, supporting a Yes alternative under the investigation-or-referral stem rather than universal referral for all pain [25]. No uniquely justified reversal was established for RF1/RF2; the core score excludes all three negatively keyed items. Full qualifications appear in File S10.

Awareness sums one point per keyed response: total 0–20, core 0–17, and contextual 0–3; non-keyed and Not sure responses score zero. Higher scores indicate key agreement. Screening ratings from Never to Always score 0–4 across 20 items (0–80), indicating frequency rather than competence. Confidence is a single 1–5 item. Scope ratings (Strongly Disagree = 1 to Strongly Agree = 5) form separate means for three core items (assessment/diagnosis, specialist referral, discharge), three investigation items (radiographs, MRI/CT, laboratory tests), and two treatment-authority items (medication, injections); certificate authority remains standalone. Seven stakeholder ratings form a support mean (Strongly Opposed = 1 to Strongly Supportive = 5); postgraduate training, increased continuing professional development, and credentialing ratings form a preparation-need mean (1–5). Eleven barriers (Not a barrier = 0 to Critical = 4) sum to 0–44. Higher values indicate stronger endorsement, support, need, or barrier intensity. No overall readiness score was calculated (File S4, Table S16b).

The principal models included numerical age (centred), sex, postgraduate academic qualification (Master’s, PhD, or DSc), musculoskeletal/sports practice, formal screening training, and prior direct-access exposure. Formal training included entry-level, postgraduate/specialty, or external CPD training, excluding self-directed study alone (File S4, Table S14). Earlier documents support clinical relevance and parsimony, but selection timing relative to the first examination of outcome associations could not be verified. The models are exploratory.

Of 12 demographic/professional domains, nationality was constant within the Saudi cohort. Region, sector, and certification involved sparse categories and additional coefficients; six certification types would add six slopes. Entry-level DPT included eight participants. Experience overlapped strongly with age and required five coefficients for six categories compared with one for numerical age. These are parsimony and measurement considerations—not missing-data exclusions or evidence that age is clinically superior. Sensitivity specifications are in File S4, Section S17.

### 2.5. Data Management

During revision, reconciliation with the original export restored 59 certification entries incorrectly represented as blank to recorded “None” responses, including 55 Saudi responses. No eligible certification responses were missing. Four named-certification/None co-selections were retained without excluding participants. This was a processing correction rather than imputation. Scored items were complete; no imputation was used. Coding rules, source checks, and de-identification procedures are documented in File S4, Sections S17 and S20, and File S9. Code standardised text encoding and response labels with checks against silent miscoding. Exact timestamps were removed from the shared data, leaving study identifiers and calendar dates.

The author manually checked age against post-licensure experience and highest qualification. No inconsistency requiring exclusion was identified, and no participant was excluded on this basis. Formal identical-pattern and low-engagement checks were not performed; such patterns alone would not establish duplicate participation.

### 2.6. Statistical Analysis

Descriptive analyses used counts, percentages, means/SDs, and selected medians/IQRs. Proportions used 95% Wilson intervals. The primary outcome used logistic regression with Wald intervals; core-scope endorsement used linear regression with HC3 standard errors and residual t intervals. Intervals were nominal 95% rather than multiplicity-adjusted. Paired scope comparisons used Wilcoxon tests and rank-biserial correlation. Spearman correlations were exploratory and interpreted primarily by magnitude; unadjusted *p* values are reported in File S4, Table S20a, with scoring-sensitivity correlations in Table S19d.

Saudi/non-Saudi comparisons used Welch or Fisher’s exact tests within an exploratory 18-outcome Holm family. Separate Holm families covered six coefficients per principal model and five awareness comparisons. Final families were refined during analysis. Post-collection revisions concerned the four-vignette outcome definition, scoring, and Saudi-primary plan; administered vignettes were unchanged, and the exact date of single-primary-outcome designation is unknown. Model diagnostics and Firth sensitivity analysis with profile-likelihood intervals [26] are reported in File S4, Tables S10–S12. Internal consistency and item-rest statistics do not validate the questionnaire or clinical keys.

Post hoc analyses pooled all 143 eligible respondents, added or substituted experience, and expanded background adjustment. Expanded models encountered separation; comparisons therefore used mean bias reduction (brglm2 version 1.1.0), nominal Wald intervals, and exploratory domain-level Holm tests. Corrected certification selections, including contradictory co-selections, were retained. The principal model had 54 participants in the smaller outcome category for six slopes (nine per coefficient); bias reduction does not remove limited information. Full specifications are in File S4, Sections S17 and S18.

Scoring sensitivity tested V7 accepting Options 2/3, V8 accepting Option 3 alone, both together, RF20 keyed Yes, and the 17-item core awareness score. Not sure remained zero; graded and Jette-compatible scores were unchanged. Subgroup and nationality comparisons retained their within-scenario Holm families; Spearman confidence correlations and paired changes used nominal percentile 95% intervals from 5000 participant bootstrap resamples. No across-scenario adjustment was applied. Exploratory checks, exact methods, and code appear in File S4, Section S19, and Files S9–S11.

Frequent screening was the unweighted mean of Often/Always percentages across 17 core items: the total Often/Always responses divided by 131 × 17, multiplied by 100. With complete data, this also equals the mean proportion of features checked per respondent. The recognition-minus-screening difference used unrounded values and compares distinct constructs, not an audited practice deficit.

Analyses and figures used R 4.6.1 [27], ggplot2 version 4.0.3 [28], and documented inference packages [29,30]. Files S2, S3 and S9–S11 provide executable code, package versions, diagnostics, and execution records.

### 2.7. Ethics

The Majmaah University Research Ethics Committee approved the study on 10 May 2026 (MUREC registration HA-01-R-088; MUREC-May.10/COM-2026/607, valid to 10 May 2027) before recruitment. Participation was voluntary; electronic consent preceded study questions. The consent wording is reproduced in File S5.

### 2.8. Data and Code Availability

The de-identified data, administered questionnaire, reproducible R code, detailed results, coding audit, and post hoc appraisal are supplied in Files S1–S11; the Supplementary Materials statement identifies each file.

### 2.9. Generative Artificial Intelligence Disclosure

ChatGPT (GPT-5.6 Sol initially, then GPT-6 Astra; OpenAI, San Francisco, CA, USA) assisted with language and readability editing, including rephrasing author-written text; document formatting; consistency checks; preparing and checking R analysis code; and preparing submission-support materials. During revision, it also assisted with locating and summarising clinical guidance for the post hoc appraisal, the English translation of Arabic open-text responses and review of comment-to-theme assignments within the predefined themes. The author reviewed the outputs and corrected coding concerns. All statistical estimates were computed using R code reviewed, executed, and validated by the author, who was solely responsible for the study design, statistical methods, coding decisions, and interpretation of results. The author reviewed and verified the final manuscript and takes full responsibility for its content.

## 3. Results

### 3.1. Participant Flow and Characteristics

**Figure 1:**
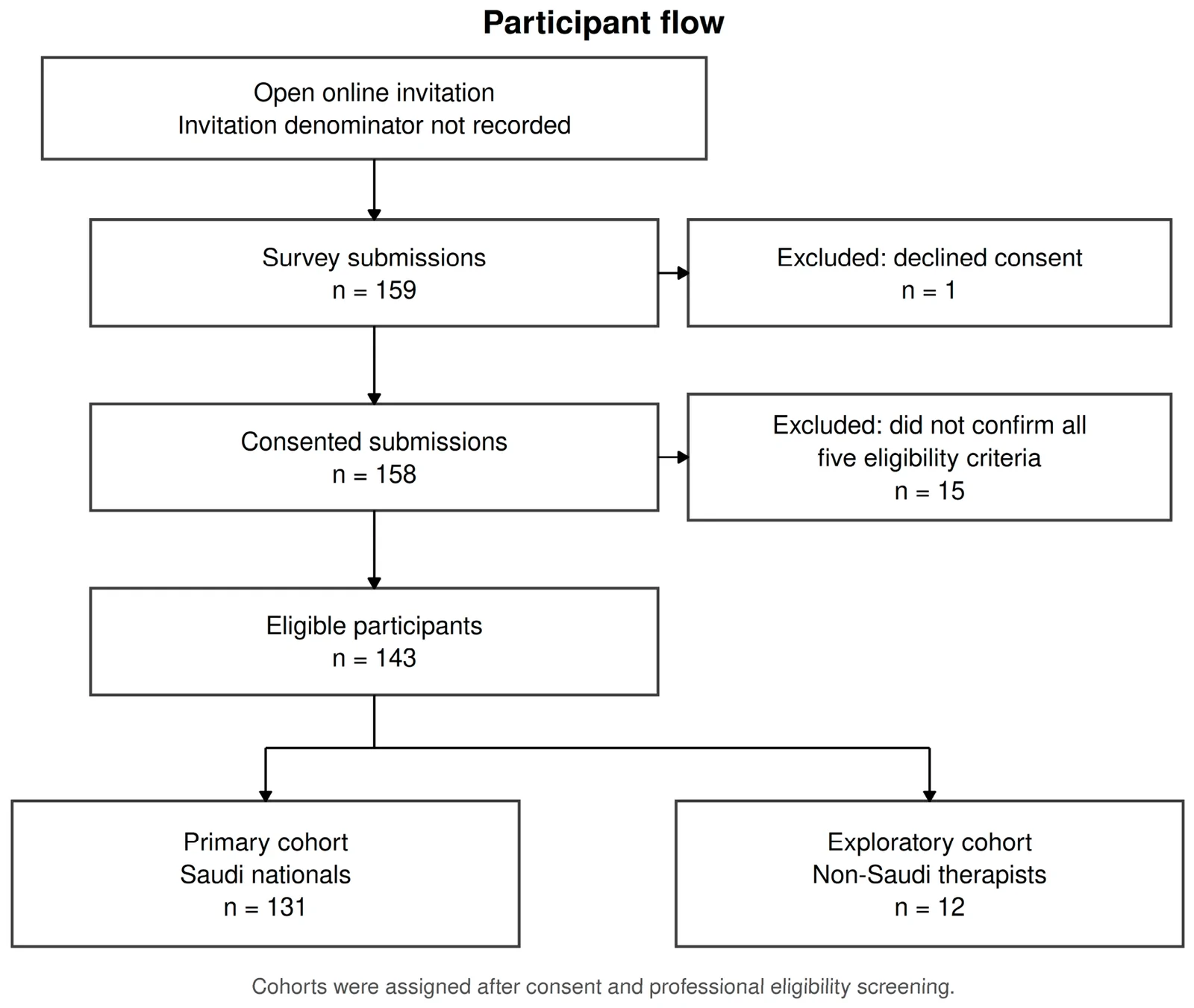
Participant flow, generated in R from consent, eligibility, and nationality responses. The invitation denominator was not recorded, so a conventional response rate cannot be calculated.

Of 159 submissions, one declined consent and 15 did not confirm all five professional-eligibility criteria; neither group provided subsequent questionnaire data. The 143 eligible respondents comprised 131 Saudi nationals in the primary cohort and 12 non-Saudi therapists in the exploratory cohort (Figure 1).

Saudi participants had a mean age of 34.2 years (SD 7.0; range 23–59), and 61.8% were men. Twelve regions were represented; 32.1% practised in Riyadh, 42.7% worked in Ministry of Health facilities, 41.2% held a Masters, PhD, or DSc, and 67.2% reported prior direct-access exposure. Although 17/131 (13.0%) selected “No formal training”, 25/131 (19.1%) lacked formal training under the analytical definition, which excludes self-directed study alone. Table 1 presents the full characteristics and training categories.

Entry-level degree and highest qualification were separate items: eight reported DPT entry, seven DPT as highest qualification, and two Other. One DPT-entry/Bachelor’s-highest discrepancy could not be resolved; both responses were retained. Table 1 separates DPT and Other.

### 3.2. Primary Safety-Related Decision Outcome

**Figure 2:**
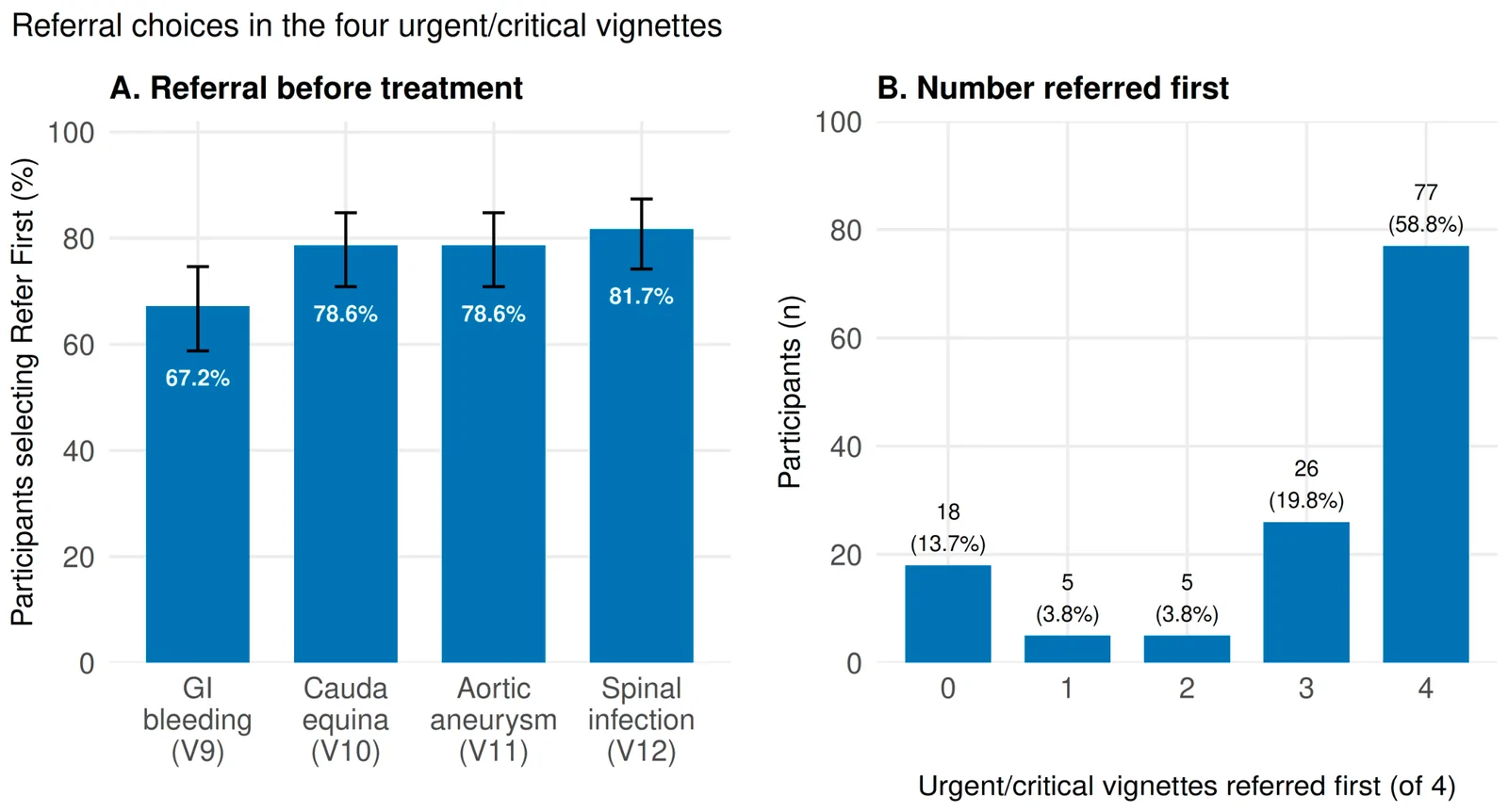
Referral choices in urgent/critical vignettes (*n* = 131). (**A**) referral-first proportions with 95% Wilson intervals. (**B**) counts and percentages by number of vignettes referred first. Uniform colour carries no safety classification.

Seventy-seven participants met the four-vignette criterion (58.8%; 95% CI 50.2–66.8). Among the remaining 54, 36 (27.5% of 131) included referral in every vignette but would begin treatment while arranging referral in at least one; 18 (13.7%) selected treatment without referral at least once, including nine for all four. The distribution of referral-first choices is shown in Figure 2; complete response patterns are in File S9, output 37. These hypothetical choices are not an observed clinical error rate.

Referral-first choices ranged from 67.2% for possible gastrointestinal bleeding to 81.7% for spinal infection (Figure 2).

### 3.3. Associations with Participant Characteristics

Postgraduate academic qualification was associated with higher odds of meeting the primary outcome (adjusted OR 4.14, 95% CI 1.63–10.51; Holm *p* = 0.017), and older age was associated with lower odds (OR 0.92/year, 95% CI 0.86–0.98; Holm *p* = 0.047). The other four coefficients did not meet the Holm threshold (Table 2 and Supplementary Figure S1).

Postgraduate qualification was also associated with stronger core-scope endorsement (adjusted mean difference 0.70, 95% CI 0.25–1.15; Holm *p* = 0.014). The small negative age association and other coefficients did not meet the Holm-adjusted threshold (Table 2).

The age functional-form check did not detect nonlinearity (*p* = 0.360); the maximum variance inflation factor was 1.34. Apparent discrimination was modest (AUC 0.685; Brier score 0.216) without predictive validation. Firth estimates were similar: postgraduate OR 3.78 (95% profile-likelihood CI 1.59–9.77) and age OR 0.92/year (0.86–0.98). Full diagnostics appear in File S4, Tables S10–S12.

Age and experience were strongly correlated (Spearman ρ = 0.894). Adding experience in a post hoc mean bias-reduced model gave an age OR of 0.85 per year (nominal 95% Wald CI 0.73–0.98). Neither this model nor the model replacing age with experience yielded a domain association surviving its exploratory Holm adjustment (File S4, Table S17b). Experience substitution/addition and expanded adjustment showed model dependence and reduced precision. With 40 slopes, the expanded model gave postgraduate OR 3.68 (95% CI 0.97–13.97) and age OR 0.90 (0.75–1.08); no expanded-model domain association survived Holm adjustment. Full estimates and limitations are in File S4, Section S17, and File S9.

Entry-level DPT participants (*n* = 8) had higher awareness scores than Bachelor-entry participants (difference 2.86, 95% CI 1.64–4.09; Holm *p* < 0.001), which was the only awareness subgroup contrast meeting the adjusted threshold. This comparison is exploratory (File S4, Table S6).

### 3.4. Clinical Decisions Across All Vignettes

The mean strict-key agreement was 7.83/12 (65.3%; SD 2.48), and graded scoring averaged 18.02/24 (SD 4.25). Table 3 presents all vignette responses and baseline keys. V7 agreement was 28.2%, although 62.6% selected referral first; accepting Options 2/3 increased agreement to 8.46/12 (70.5%). Submitted keys remain the baseline. Additional summaries and alternative-score analyses are in File S4, Tables S8 and S15 and Sections S19 and S20.

Jette-compatible category percentages were 88.2% for musculoskeletal, 91.6% for medically concerning, and 76.5% for urgent/critical vignettes. These are contextual comparisons rather than evidence of instrument equivalence (File S4, Table S8).

### 3.5. Red-Flag Awareness, Screening, and Confidence

**Figure 3:**
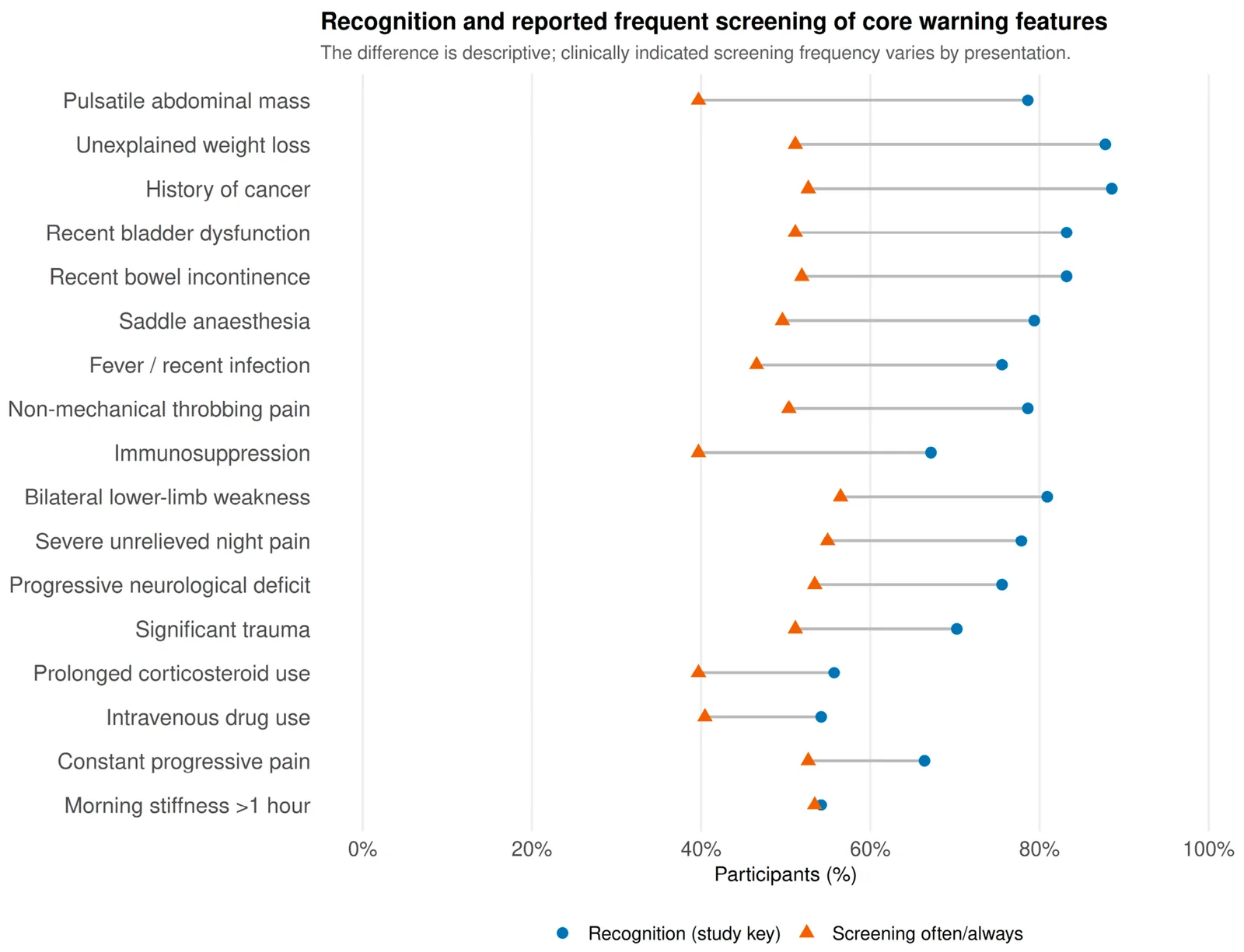
Recognition and reported frequent screening for 17 core warning features (*n* = 131). Circles denote recognition; triangles denote Often/Always screening, as identified in the legend. Connecting lines show descriptive differences—not audited screening deficits.

The mean total awareness was 13.94/20 (SD 3.89; 69.7%), core awareness 12.57/17 (SD 3.96; 74.0%), and contextual-item agreement 1.37/3 (45.5%). Recognition was highest for cauda equina/neurological and malignancy/systemic domains (80.5% and 80.2%), and it was lower for fracture and the inflammatory item (63.0% and 54.2%). For failure to improve after more than four weeks, 17.6% answered No; this is descriptive because the stem combined investigation and referral. Separate Yes, No, and Not sure counts appear in File S4, Table S18b; Figure 3 shows core items.

The screening-frequency score averaged 47.31/80 (SD 24.94). Across 17 core items, 1094/2227 screening responses indicated Often/Always (49.1%) compared with 1647/2227 Yes recognition responses (74.0%). These are item-level summaries—not percentages of participants classified as frequent screeners. Their difference, (1647 − 1094)/2227 × 100, was 24.8 percentage points after rounding. The awareness–screening correlation was weak (Spearman ρ = 0.28). Exact calculations are in File S4, Section S18, and File S9.

Confidence averaged 3.76/5 (SD 0.86) with 66.4% rating 4 or 5. Its exploratory correlations with awareness, strict-key agreement, and referral-first counts were weak (Spearman ρ = 0.26, 0.28, and 0.24, respectively; File S4, Section S20).

### 3.6. Regulation, Scope, Support, Education, and Barriers

The four regulatory interpretations were limited-settings permission, 60/131 (45.8%; 95% CI 37.5–54.3); full permission, 27/131 (20.6%); no permission, 32/131 (24.4%); and unsure, 12/131 (9.2%). The first represents the investigator’s interpretation, which is not verified legal knowledge. Self-rated regulatory awareness was full in 35.1% and partial in 50.4%.

**Figure 4:**
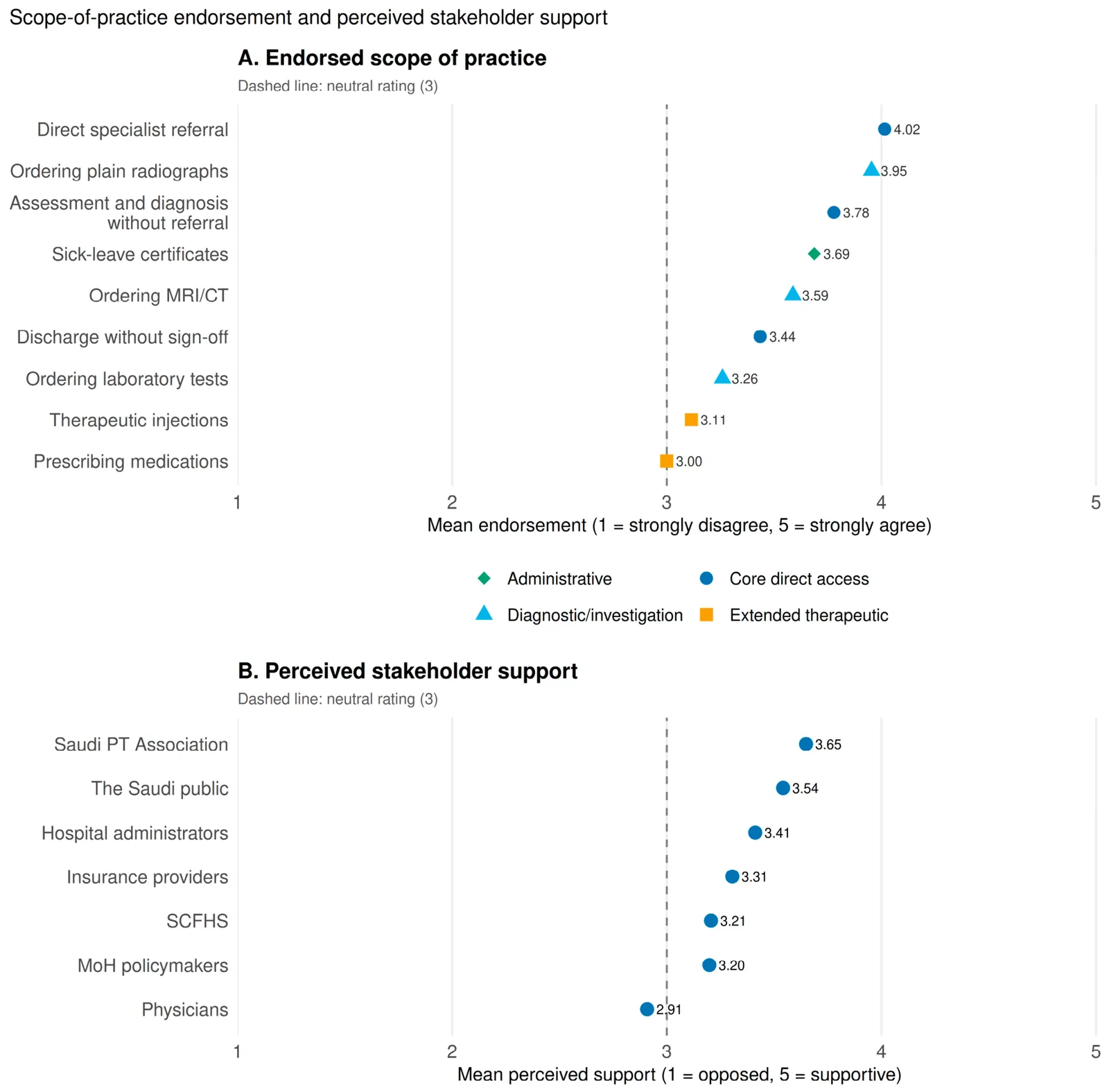
Mean scope endorsement (**A**) and perceived stakeholder support (**B**). Scope domains are identified by both colour and shape in the legend. Dashed lines mark neutral ratings (3) on 1–5 scales. Support points use one colour. CT, computed tomography; MRI, magnetic resonance imaging; MoH, Ministry of Health; PT, physical therapy; SCFHS, Saudi Commission for Health Specialties.

Core-scope endorsement averaged 3.74/5 (SD 1.11) compared with 3.06 (SD 1.27) for medication/injection authority (Wilcoxon *p* < 0.001; rank-biserial correlation 0.64). Direct specialist referral (72.5% agreement), radiograph ordering (71.0%), and assessment/diagnosis without prior referral (67.9%) received the highest endorsement; prescribing received the lowest (35.9%; Figure 4A).

Physicians were perceived as the least supportive group (30.5% support, 39.7% opposition). The Saudi Physical Therapy Association (58.0% support) and public (51.9%) were viewed more favourably (Figure 4B).

Only 34.4% considered entry-level education adequate preparation for direct access (mean 2.93, SD 1.30); 68.7%–71.0% supported increased CPD, postgraduate specialty training, or credentialing. The preparation-need score averaged 3.90/5 (SD 1.12).

**Figure 5:**
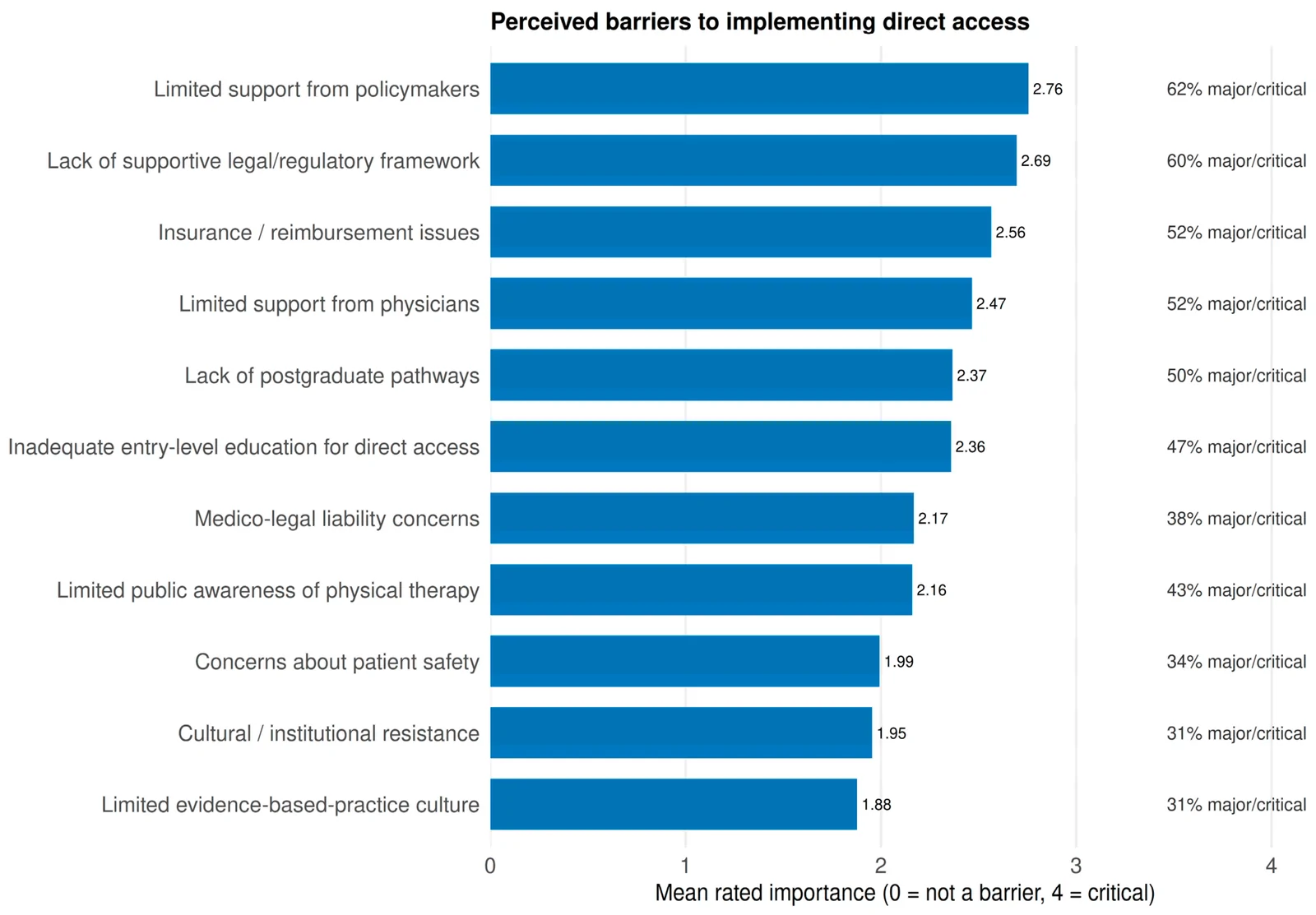
Perceived barriers, ranked by mean rating (0 = not a barrier; 4 = critical). Bar-end labels show means; right-hand labels show percentages rating each barrier major/critical. All bars represent the same measure.

The highest-rated barriers were limited policymaker support (mean 2.76; 61.8% major/critical), absence of a supportive legal framework (2.69; 60.3%), reimbursement limitations (2.56; 51.9%), and limited physician support (2.47; 51.9%). Patient safety ranked ninth (1.99; 33.6%); the overall barrier score averaged 25.36/44 (SD 9.71; Figure 5).

### 3.7. Additional Analyses, Scoring Sensitivity, and Measurement Properties

Thirty-two substantive comments (28 Saudi, four non-Saudi) were coded. Training/differential diagnosis appeared in 22/32 (68.8%), referral pathways/collaboration in 10/32 (31.3%), and regulation/scope/accountability in 9/32 (28.1%). Multiple labels were allowed. File S8 reports all eight themes and comment-level assignments; percentages use these 32 comments as the denominator.

Pooling all eligible participants gave 83/143 meeting the primary criterion (58.0%; 95% CI 49.8–65.8) compared with 58.8% in the Saudi cohort. No nationality comparison survived Holm adjustment; the 12-person non-Saudi group limits inference and does not establish equivalence (File S4, Tables S13a,b and S18a).

Internal-consistency estimates and vignette item-rest correlations are reported in File S4, Table S20a, and File S9. These descriptive statistics do not establish broader reliability, questionnaire validity, or clinical key validity.

Under the tested alternative keys, confidence associations remained weak. For vignette agreement, Spearman ρ was 0.275 under the submitted key and 0.259 with both V7/V8 alternatives; the paired change was −0.017 (95% bootstrap CI −0.062 to 0.030). All paired correlation-change intervals included zero, which does not establish equivalence. The DPT awareness contrast remained positive and met the within-scenario Holm threshold, while other subgroup intervals included zero. Full scenario estimates, effect sizes, and uncertainty are in File S4, Section S19, and File S9.

These scoring scenarios left V9–V12 and the six predictors unchanged, so verification refits produced identical main-model estimates and standard errors. This expected invariance does not test alternative keys for the primary outcome.

## 4. Discussion

### 4.1. Principal Findings

The survey identified differences across clinical decisions, recognition, reported screening, and implementation perspectives. Most respondents selected referral before treatment in all four urgent/critical vignettes, while other choices included both concurrent referral and treatment without referral. Recognition exceeded reported screening, regulatory views differed, and policy and professional-support barriers were prominent. Qualification and age associations were exploratory and less precise under broader adjustment.

### 4.2. Patient Safety and Clinical Decision-Making

Jette-compatible scores provide descriptive context, but different vignettes, populations, and settings prevent direct inferential comparison [3]. Vignette choices neither establish observed clinical performance nor estimate errors in practice (File S4, Table S8).

Other vignette studies report a variable management of serious presentations. Danish findings associated experience and practice in a quality-audited clinic with appropriate critical-vignette decisions [31]; Austrian performance was lower for critical than musculoskeletal vignettes [32]. French findings distinguished appropriate management from the identification of the diagnostic hypothesis [33]. These comparisons reinforce the difference between referral and diagnostic labelling. The observed association with postgraduate qualification cannot establish an educational effect. Age may reflect experience, training-era differences, selection, or residual confounding. Its simpler numerical measurement supports parsimony rather than clinical superiority; sensitivity analyses do not resolve these explanations.

V7 demonstrates the distinction between preferred-key agreement and safety: most respondents chose referral first, which the strict key did not credit. Neither the post hoc appraisal nor the negative item-rest correlation establishes a uniquely optimal treatment/referral sequence. V8 is similarly abbreviated. Retaining submitted keys preserves the scoring history, but it does not provide a claim of prospective specification or superior validity. Tested alternatives changed score levels but had small effects on confidence associations; they did not validate the questionnaire or stress-test the primary referral rule (File S10; File S4, Section S19).

### 4.3. Red-Flag Knowledge, Screening, and Confidence

Core recognition may partly reflect a tendency to answer Yes. The compound investigation-or-referral wording also prevents interpreting negatively keyed responses as standalone referral discrimination or overcalling. The recognition–screening difference compares keyed answers with recalled initial-assessment frequency without a recall period or patient-specific indication. It is not an audited deficit. Earlier Saudi findings provide context but use different items and thresholds [5]. RF20 and core-only analyses yielded similar secondary association patterns, while uncertainty about wording and thresholds remains.

Weak confidence correlations indicate that self-perceived confidence should not substitute for a contextual assessment of clinical reasoning and referral decisions. The exploratory DPT awareness association requires particular caution because only eight participants were in this subgroup.

### 4.4. Regulation, Scope, and Implementation Context

Regulatory responses represent differing interpretations—not a demonstrated knowledge deficit. Licensing provisions for private centres and referral requirements for government-hospital and specified home-care services concern different settings [13,14,15,34]. They do not establish a national self-referral standard; undated annexes also limit the confirmation of applicability throughout collection. File S4, Table S16d identifies exact provisions, versions, dates, and limitations. Core access should remain distinct from extended diagnostic or treatment privileges.

Policy, reimbursement, physician support, and organisational barriers accord with regional evidence [6,7]. The low relative ranking of perceived patient-safety concerns and the four-vignette criterion assess different constructs. Concurrent referral may be appropriate depending on stability, intervention, and timing, which were not recorded; immediate escalation remains essential when indicated. These findings should not be generalised across differing regulatory systems.

### 4.5. Implications and Future Research

This study did not test implementation or credentialing. A scoping review identified 17 first-contact competencies across five domains [12], and a French Delphi study identified 27 across five domains [35]. Such frameworks could inform a multidisciplinary development of Saudi criteria spanning clinical reasoning, communication, collaboration, and professional practice. A qualification, confidence rating, or survey threshold alone is insufficient. Staged pilots should define eligibility, referral/escalation, communication, medical-review access, reimbursement, liability, and safety monitoring.

A systematic review of 21 studies involving 90,401 patients found generally lower general-practitioner use and often lower imaging or medication use with non-inferior outcomes and neutral or lower costs; certainty was mostly low or very low [36]. Qualitative research emphasised communication, scope definition, and resources [37]. Saudi pilots should assess referral appropriateness, delayed diagnosis, adverse events, access, healthcare use, outcomes, costs, and equity. Larger studies should include formal Saudi validation, cognitive interviewing, test–retest assessment, audited decisions, educational interventions, and stakeholder research. Comparable Gulf studies could clarify regional patterns.

### 4.6. Strengths and Limitations

Strengths include a concurrent assessment of distinct clinical, behavioural, and implementation domains, participant-level referral choices, uncertainty estimates, multiplicity adjustment, and sensitivity analyses. The supplied de-identified data, questionnaire, scoring specifications, and executable code support transparency and reproducibility.

The cross-sectional convenience sample cannot establish causality or national representativeness. Unknown invitation and dissemination denominators preclude a response rate. Recruitment fell short of 385; the 131-person primary cohort yielded a 16.6-percentage-point-wide confidence interval for the primary proportion. Interest in direct access may have influenced participation, and nearly one third practised in Riyadh. Small non-Saudi and DPT groups limit exploratory comparisons; pooled results do not remove possible confounding by education or previous practice context.

Formal questionnaire validity, test–retest reliability, and independent prelaunch or post hoc clinician appraisal were not established. Internal consistency does not remedy these gaps. The guideline appraisal was targeted and conducted with keys/results available; exact-key judgments remain investigator interpretations, and sensitivity checks cover selected alternatives only. Eligibility was self-reported; English-only administration may have affected participation or comprehension. Duplicate participation could not be established. Vignettes omit clinical context, workplace rules may influence answers, and unaudited screening, confidence, and attitudes are susceptible to reporting bias.

Sparse categories, limited outcome information, residual confounding, and multiple analyses constrain interpretation. The expanded models exhibited separation and wider intervals despite bias reduction; age and experience overlapped strongly. Final outcome, scoring, cohort, and multiplicity decisions were not all prospectively documented, and predictor-selection timing remains unverified. Holm adjustment cannot remove these limitations. Regulatory agreement is investigator-defined, not a national knowledge standard; the findings support hypotheses and prospective evaluation rather than judgments about clinicians or workforce safety.

Open-text findings derive from 32 self-selected comments within eight predefined themes. The author reviewed and corrected labels during revision with AI assistance without an independent coder or translation check. Ambiguous-comment interpretations remain subjective, and theme frequencies neither measure importance nor estimate population prevalence (File S8).

## 5. Conclusions

This exploratory survey can inform the evaluation of professional preparation, regulatory clarification, and setting-specific referral pathways for direct-access physical therapy. Its vignettes and self-reported practices do not establish observed clinical safety, causal effects of qualifications, or national workforce readiness. In particular, not meeting the strict four-vignette criterion includes concurrent referral and is not a clinical error rate. Prospective evaluation should distinguish core access from extended privileges and assess referral appropriateness, escalation, service outcomes, and patient safety.

## Figures and Tables

**Table 1:** Characteristics of the primary analytic cohort (Saudi nationals, *n* = 131).

Characteristic	Value
Age, years—mean (SD)	34.2 (7.0)
Age, years—median [IQR]	33.0 [29.5–38.0]
Sex	
Male	81 (61.8%)
Female	50 (38.2%)
Region of practice	
Riyadh	42 (32.1%)
Eastern Province	15 (11.5%)
Makkah	14 (10.7%)
Asir	11 (8.4%)
Qassim	10 (7.6%)
Other represented regions (7)	39 (29.8%)
Practice sector	
Ministry of Health	56 (42.7%)
Private hospital or clinic	35 (26.7%)
Military/Interior/National Guard	16 (12.2%)
Academic or rehabilitation centre	12 (9.2%)
Other	12 (9.2%)
Entry-level degree	
Bachelor of Physical Therapy	123 (93.9%)
Doctor of Physical Therapy	8 (6.1%)
Highest qualification	
Bachelors	68 (51.9%)
Masters	44 (33.6%)
PhD/DSc	10 (7.6%)
Doctor of Physical Therapy	7 (5.3%)
Other	2 (1.5%)
Post-licensure experience	
1 to <3 years	24 (18.3%)
3 to <6 years	23 (17.6%)
6 to <10 years	34 (26.0%)
10 years or more	50 (38.2%)
Primary clinical area	
Musculoskeletal/orthopaedic	55 (42.0%)
General or mixed practice	30 (22.9%)
Neurological	16 (12.2%)
Paediatric	13 (9.9%)
Sports	9 (6.9%)
Other	8 (6.1%)
Medical-screening training (multiple response)	
Postgraduate/specialty training	69 (52.7%)
Entry-level education	46 (35.1%)
External CPD course or workshop	42 (32.1%)
Self-directed study	41 (31.3%)
No formal training	17 (13.0%)
Prior direct-access exposure	
Currently practising under direct access	36 (27.5%)
Limited/partial within Saudi Arabia	46 (35.1%)
Previously outside Saudi Arabia	6 (4.6%)
Never	43 (32.8%)

Percentages use *n* = 131 unless otherwise stated; medical-screening training was a multiple-response item and therefore does not sum to 100%. For analytical models, formal training required entry-level education, postgraduate/specialty training, or an external CPD course/workshop; self-directed study alone was excluded. Abbreviations: CPD, continuing professional development; IQR, interquartile range; SD, standard deviation.

**Table 2:** Adjusted associations with referral before intervention for all four urgent/critical vignettes and core scope endorsement among Saudi physical therapists (*n* = 131).

Outcome/Predictor	Estimate	95% CI	*p*	Holm *p*	Reference/Increment
**Referral before intervention for all four urgent/critical vignettes (logistic model)**					
Postgraduate academic qualification	4.14	1.63–10.51	0.003	0.017	Other qualifications
Age, per additional year	0.92	0.86–0.98	0.009	0.047	Per additional year
Female sex	1.51	0.69–3.30	0.306	1.000	Male
MSK/sports practice	0.72	0.33–1.57	0.413	1.000	Other clinical areas
Any formal screening training	0.78	0.30–2.06	0.619	1.000	No formal training
Any prior direct-access exposure	0.82	0.37–1.82	0.624	1.000	No prior exposure
**Core scope endorsement (linear model)**					
Postgraduate academic qualification	0.70	0.25–1.15	0.002	0.014	Other qualifications
Age, per additional year	−0.035	−0.068–−0.003	0.032	0.161	Per additional year
Female sex	0.21	−0.20–0.62	0.319	0.957	Male
MSK/sports practice	−0.19	−0.59–0.21	0.351	0.957	Other clinical areas
Any formal screening training	0.21	−0.36–0.78	0.470	0.957	No formal training
Any prior direct-access exposure	0.27	−0.15–0.70	0.207	0.829	No prior exposure

Estimates are adjusted odds ratios or adjusted mean differences. Reference categories are explicit. Logistic intervals are nominal 95% Wald intervals; linear intervals use HC3 standard errors and residual t inference. Holm adjustment covers six coefficients per model, not confidence intervals. Formal training excludes self-directed study alone. CI, confidence interval; MSK, musculoskeletal.

**Table 3:** Management decisions for the twelve clinical vignettes (Saudi cohort, *n* = 131).

Vignette	Baseline Key	Option 1 *n* (%)	Option 2 *n* (%)	Option 3 *n* (%)	Strict-Key % [95% CI]
V1 Lateral elbow pain	1	92 (70.2)	29 (22.1)	10 (7.6)	70.2 [61.9–77.4]
V2 Patellofemoral pain	1	87 (66.4)	33 (25.2)	11 (8.4)	66.4 [58.0–73.9]
V3 Mechanical low-back pain	1	85 (64.9)	33 (25.2)	13 (9.9)	64.9 [56.4–72.5]
V4 Capsular shoulder stiffness	1	52 (39.7)	54 (41.2)	25 (19.1)	39.7 [31.7–48.3]
V5 Plantar heel pain	1	84 (64.1)	30 (22.9)	17 (13.0)	64.1 [55.6–71.8]
V6 Cervical radicular pain	1	67 (51.1)	47 (35.9)	17 (13.0)	51.1 [42.7–59.5]
V7 Progressive diabetic neuropathy	2	12 (9.2)	37 (28.2)	82 (62.6)	28.2 [21.2–36.5]
V8 Shoulder pain with palpitations	2 or 3	10 (7.6)	34 (26.0)	87 (66.4)	92.4 [86.5–95.8]
V9 Possible GI bleeding with NSAID use	3	14 (10.7)	29 (22.1)	88 (67.2)	67.2 [58.7–74.6]
V10 Cauda equina syndrome	3	11 (8.4)	17 (13.0)	103 (78.6)	78.6 [70.8–84.8]
V11 Abdominal aortic aneurysm	3	13 (9.9)	15 (11.5)	103 (78.6)	78.6 [70.8–84.8]
V12 Spinal infection	3	10 (7.6)	14 (10.7)	107 (81.7)	81.7 [74.2–87.4]

Option 1: treat without referral; Option 2: treat and consult/refer; Option 3: refer before intervention. Entries are *n* (%) of 131; intervals are 95% Wilson intervals. The baseline is the submitted study-defined key, not an independently validated standard. V1–V6 are musculoskeletal, V7–V8 medically concerning, and V9–V12 urgent/critical; V8 accepts Options 2/3. Post hoc alternatives appear in File S4, Section S19, and Files S10 and S11. GI, gastrointestinal.

## Data Availability

The de-identified dataset, complete questionnaire, reproducible R code, detailed results, and coding and appraisal records are provided in Supplementary Files S1–S11, as listed above.

## Supplementary Materials

The following supporting information can be downloaded at https://www.mdpi.com/article/10.3390/healthcare14193347/s1. Files S1–S11 contain the supporting information; the accompanying contents list identifies the files and their items. The clinical sources used in File S10 are included in the article reference list [4,8,19,20,21,22,23,24,25,38,39,40,41,42,43,44].

## Abbreviations

DPT	Doctor of Physical Therapy
OR	odds ratio
