# Supplementary material for: Clinical Decision Making, Red-Flag Recognition, and Scope-of-Practice Perspectives for Direct Access Physical Therapy in Saudi Arabia
Source: Healthcare (Basel). 2026 Oct 8;14(19):3347. doi: 10.3390/healthcare14193347 (PMC13664920; doi:10.3390/healthcare14193347)
Supplement: Supplementary file 1 [file healthcare-14-03347-s001.zip › File_S10_Post_Hoc_Guideline_Based_Appraisal.pdf]

Supplementary File S10

Post hoc guideline based appraisal of the clinical questionnaire

This post hoc appraisal documents the clinical support and uncertainty behind the questionnaire keys used in the direct-access physical therapy survey. It was prepared during manuscript revision on 26–27 September 2026, after the original results were known. It supports interpretation and motivates exploratory scoring sensitivity analyses; it is not independent expert validation.

The appraisal compares each clinical item with relevant professional guidelines, a consensus position paper or a clinical framework. The mappings and alternative keys are investigator interpretations of those sources. AI assisted source identification, synthesis and preparation under author oversight. No independent clinician panel, agreement assessment or formal evidence grading was performed. The administered questionnaire and participant responses remain unchanged.

Support labels are descriptive: Clear means the relevant clinical action or concern is directly supported; Conditional means context affects the response; Uncertain means the exact study key or threshold is not established by the guidance. These labels are not GRADE ratings, probabilities of correctness or validation results. A key retained without an alternative test is not thereby validated.

Original means the submitted scoring used as the analysis baseline, not a claim of prospective specification. Sources are listed by the versions consulted; current guidance used in this revision is not evidence of what informed the original questionnaire. Complete wording is in File S5, baseline scoring in File S4, sensitivity results in Files S4 and S9, and executable sensitivity code in File S11.

Clinical vignettes

Options: 1 = PT without medical referral; 2 = PT with concurrent consultation/referral; 3 = medical referral before any PT. These are broad categories. Guidelines support clinical decisions but do not validate these exact option boundaries. V9–V12 form the primary four-vignette referral-first composite. The urgency and destination below are post hoc clinical applications, not additional response options presented to participants.

Item and original strict key	Guideline support and uncertainty	Alternative and sensitivity test
V1<br>Lateral elbow pain<br>Key 1	Conditional. The lateral elbow guideline supports PT assessment and treatment for this presentation [1]. It does not establish that medical consultation is incorrect.	Option 2 can be reasonable if assessment or workplace rules require consultation. No alternative tested.
V2<br>Anterior knee pain<br>Key 1	Conditional. The patellofemoral pain guideline supports exercise-based PT for compatible symptoms [2]. The brief vignette does not establish a definitive diagnosis.	Consultation can be reasonable if diagnostic uncertainty remains. No alternative tested.
V3<br>Acute mechanical back pain<br>Key 1	Conditional. Conservative PT is supported when serious causes are not suspected [3]. The stated presentation supports this choice, without proving referral is inappropriate.	Consultation depends on assessment and local rules. No alternative tested.
V4<br>Capsular shoulder stiffness<br>Key 1	Conditional. The adhesive capsulitis guideline supports PT for compatible mobility deficits [4]. Medical co-management is also compatible with that guideline.	Option 2 is plausible for diagnostic review or additional treatment. No alternative tested.
V5<br>Plantar heel pain<br>Key 1	Conditional. Typical first-step pain and local tenderness support PT management under the plantar heel pain guideline [5].	Consultation remains possible if another cause is suspected. No alternative tested.

Item and original strict key	Guideline support and uncertainty	Alternative and sensitivity test
V6<br>Neck and arm pain<br>Key 1	Conditional. Conservative care is supported for neck pain with radiating pain after screening [6]. No progressive deficit is described.	Medical consultation can be reasonable if symptoms worsen or assessment raises concern. No alternative tested.
V7<br>Progressive foot sensory symptoms with diabetes<br>Key 2	Uncertain exact key. ADA guidance supports neuropathy assessment and exclusion of other causes [7, recommendations 12.17–12.18]. Six-month progression and overdue review support medical assessment; it does not uniquely establish treatment before versus after review.	Yes. Accept 2 or 3 in V7_accept_2_or_3. Referral first is a defensible alternative; timely primary care/diabetes review is appropriate.
V8<br>Shoulder pain with fatigue and palpitations<br>Keys 2 or 3	Uncertain exact key. EHRA supports evaluation of palpitations, including history, examination and ECG [8]. Missing cardiovascular assessment prevents deciding whether PT should precede review.	Yes. V8_refer_first_only accepts 3 alone as a stricter hypothetical rule. The source does not establish it as uniquely correct or prove 2 unsafe.
V9<br>Dark stools, NSAIDs, fatigue and tachycardia<br>Key 3	Clear referral priority. Suspected gastrointestinal bleeding supports urgent medical assessment [9, recommendations 1.1.1 and 1.3.1–1.3.2]. Application: same-day acute medical/ED assessment before routine PT; immediate escalation if unstable.	No additional scoring alternative tested. Guidance supports acute assessment, not a universal prohibition on every supportive intervention.
V10<br>Retention, saddle numbness and bilateral weakness<br>Key 3	Clear referral priority. Suspected cauda equina syndrome requires immediate referral [10, recommendation 1.7.3]. Application: emergency pathway to a service able to arrange urgent spinal assessment/MRI.	No additional scoring alternative identified for delaying emergency assessment. Key retained; no alternative tested.
V11<br>Sudden back/abdominal pain and pulsatile mass<br>Key 3	Clear referral priority. Suspected symptomatic/ruptured AAA requires immediate assessment and vascular discussion [11, recommendations 1.1.7–1.1.9]. Application: emergency transfer/ED and vascular service.	No additional scoring alternative identified for delaying emergency assessment. Key retained; no alternative tested.
V12<br>Back pain, fever, weight loss and IV drug use<br>Key 3	Clear referral priority. Suspected spinal infection warrants diagnostic work-up [12, recommendations 1 and 6–8]. Application: same-day acute medical/ED assessment with infectious disease/spinal input; immediate escalation for sepsis or neurological compromise.	No additional scoring alternative tested. Same-day destination/timing is the investigator application; IDSA does not specify a PT questionnaire option.

Warning features

The administered stem combined “further investigation or referral”. Recognising a feature as a reason to reassess is different from treating it as a sufficient referral indication. A Yes key therefore represents recognition of possible concern, not mandatory referral in every patient. All 17 core items are positively keyed, which may favour general Yes responding. No and Not sure remain distinct responses, although both score zero on these core items.

Item and original key	Guideline support and uncertainty	Alternative and sensitivity test
RF1<br>Age >50 with new back pain<br>No	Uncertain exact No key. Age contributes to risk assessment but is not a sufficient standalone referral rule [13,14].	Yes may reflect suspicion rather than referral. Excluded in Core17; no individual reversal tested.

Item and original key	Guideline support and uncertainty	Alternative and sensitivity test
RF2<br>Age <20 with new back pain<br>No	Uncertain exact No key. Age must be interpreted with the clinical presentation; no universal age-only Yes/No rule is established [13,14].	Context-dependent alternative possible. Excluded in Core17; no individual reversal tested.
RF3<br>Cancer history<br>Yes	Clear concern. Cancer history with compatible spinal symptoms warrants further assessment [15, box 1].	No alternative identified for recognising concern. Key retained; no rekeying tested.
RF4<br>Unexplained weight loss >10% in 3–6 months<br>Yes	Conditional. Unexplained weight loss raises concern, but this exact threshold is not validated here [13,14].	Exact cutoff uncertain. Key retained; no alternative tested.
RF5<br>Progressive non-mechanical pain<br>Yes	Conditional. Persistent/progressive pain can support suspicion of serious pathology; it is not diagnostic alone [13,15].	Referral depends on the full assessment. Key retained; no alternative tested.
RF6<br>Unrelieved severe night pain<br>Yes	Conditional. Night pain can raise concern, particularly with other cancer or infection features [13,15].	Referral depends on the full assessment. Key retained; no alternative tested.
RF7<br>Fever or recent bacterial infection<br>Yes	Conditional. Fever or recent infection accompanying new/worsening spinal pain supports investigation [12, recommendations 1, 3 and 5].	History alone does not define urgency. Key retained; no alternative tested.
RF8<br>IV drug use<br>Yes	Conditional. IV drug use increases concern for spinal infection in a compatible presentation [12, diagnostic evidence summary].	A risk factor, not an automatic referral rule. Key retained; no alternative tested.
RF9<br>Immunosuppression<br>Yes	Conditional. Immunosuppression increases concern for infection; symptoms and examination determine action [12, diagnostic evidence summary].	A risk factor, not an automatic referral rule. Key retained; no alternative tested.
RF10<br>Significant trauma<br>Yes	Conditional. Trauma with age/bone-health context raises fracture concern [13,14].	Assessment determines fracture suspicion and urgency. Key retained; no alternative tested.
RF11<br>Saddle/perineal numbness<br>Yes	Clear concern. New perineal sensory change with compatible spinal symptoms warrants immediate cauda equina assessment [10, recommendation 1.7.3].	No alternative identified for recognising concern. Key retained; no rekeying tested.
RF12<br>New bladder retention/overflow<br>Yes	Clear concern. New bladder dysfunction with compatible spinal symptoms warrants immediate cauda equina assessment [10, recommendation 1.7.3].	No alternative identified for recognising concern. Key retained; no rekeying tested.
RF13<br>New bowel incontinence<br>Yes	Clear concern. New bowel dysfunction with compatible spinal symptoms warrants immediate cauda equina assessment [10, recommendation 1.7.3].	No alternative identified for recognising concern. Key retained; no rekeying tested.

Item and original key	Guideline support and uncertainty	Alternative and sensitivity test
RF14<br>Bilateral leg weakness/sensory loss<br>Yes	Conditional. Pattern, onset and associated sphincter/spinal signs determine possible cord/cauda equina involvement [10,15].	Urgency cannot be inferred from this isolated phrase. Key retained; no alternative tested.
RF15<br>Progressive neurological deficit<br>Yes	Clear concern. Progressive neurological changes require assessment; the pathway depends on rate, distribution and associated signs [10].	Not every deficit has the same urgency. Key retained; no alternative tested.
RF16<br>Morning stiffness >1 hour in a younger patient<br>Yes	Conditional. An inflammatory pattern merits assessment. NG65 uses a symptom/risk-factor combination, not this isolated duration cutoff [16, recommendation 1.1.5].	Exact cutoff and automatic referral are uncertain. Key retained; no alternative tested.
RF17<br>Pulsatile abdominal mass<br>Yes	Clear concern. This finding supports assessment for AAA; symptoms determine urgency [11, recommendations 1.1.4 and 1.1.7–1.1.9].	No alternative identified for recognising concern. Key retained; no rekeying tested.
RF18<br>Non-mechanical back pain with vascular risk<br>Yes	Conditional. Back/abdominal pain with older age and vascular risk raises AAA suspicion [11, recommendation 1.1.7].	The cluster warrants assessment, not diagnosis from pain quality alone. Key retained; no alternative tested.
RF19<br>Prolonged corticosteroid use<br>Yes	Conditional. Steroid exposure contributes to fracture risk; dose, duration and other findings matter [13,14].	Not an automatic referral rule in isolation. Key retained; no alternative tested.
RF20<br>No treatment response for >4 weeks<br>No	Uncertain exact No key. The 2012 LBP guideline identifies failure to improve within about 30 days as reason for reassessment/possible referral [14, differential diagnosis]. This does not establish universal referral for all pain.	Yes tested in RF20_yes; also excluded in Core17. The broad "investigation or referral" stem makes Yes defensible.

Link to the sensitivity analyses

V7_accept_2_or_3 and V8_refer_first_only were tested separately and together (V7_and_V8_alternatives). RF20_yes reversed only RF20. Core17 omitted RF1, RF2 and RF20 rather than declaring their opposite keys correct. The nationality comparisons also examined the combined V7/V8/RF20 scenario. All alternatives were post hoc and exploratory; the original keys remain the baseline. The graded 0–24 and Jette-compatible scores were not recalibrated. V9–V12 and the six model predictors were unchanged, so these scenarios do not change the primary regression model.

These selected tests examine sensitivity to specific ambiguities; they do not exhaust every plausible alternative. Confidence, self-reported screening, scope attitudes, implementation ratings and open text have no clinical correct-answer key and are outside this appraisal. Regulatory agreement is also outside this clinical appraisal and remains agreement with the investigator interpretation, not a validated national knowledge score.

References (numbering within File S10)

1. Lucado AM et al. Lateral Elbow Pain and Muscle Function Impairments. J Orthop Sports Phys Ther. 2022;52:CPG1–CPG111. Relevant sections: diagnosis and interventions.
https://doi.org/10.2519/jospt.2022.0302

2. Willy RW et al. Patellofemoral Pain. J Orthop Sports Phys Ther. 2019;49:CPG1–CPG95. Relevant sections: diagnosis and exercise therapy.
https://doi.org/10.2519/jospt.2019.0302

3. George SZ et al. Interventions for the Management of Acute and Chronic Low Back Pain Revision 2021. J Orthop Sports Phys Ther. 2021;51:CPG1–CPG60. Relevant section: acute low back pain interventions.
https://doi.org/10.2519/jospt.2021.0304

4. Kelley MJ et al. Shoulder Pain and Mobility Deficits Adhesive Capsulitis. J Orthop Sports Phys Ther. 2013;43:A1–A31. Relevant sections: diagnosis and interventions.
https://doi.org/10.2519/jospt.2013.0302

5. Koc TA Jr et al. Heel Pain Plantar Fasciitis Revision 2023. J Orthop Sports Phys Ther. 2023;53:CPG1–CPG39. Relevant sections: diagnosis and interventions.
https://doi.org/10.2519/jospt.2023.0303

6. Blanpied PR et al. Neck Pain Revision 2017. J Orthop Sports Phys Ther. 2017;47:A1–A83. Relevant section: neck pain with radiating pain.
https://doi.org/10.2519/jospt.2017.0302

7. American Diabetes Association Professional Practice Committee for Diabetes. Retinopathy, Neuropathy, and Foot Care Standards of Care in Diabetes 2026. Diabetes Care. 2026;49(Suppl 1):S261–S276. Recommendations 12.17–12.18 and neuropathy diagnosis.
https://doi.org/10.2337/dc26-S012

8. Raviele A et al. Management of patients with palpitations a position paper from the European Heart Rhythm Association. Europace. 2011;13:920–934. Relevant section: diagnostic management.
https://doi.org/10.1093/europace/eur130

9. NICE. Acute upper gastrointestinal bleeding in over 16s management. CG141. 2012; updated 2016; linked technology-appraisal information updated 2025. Recommendations 1.1.1 and 1.3.1–1.3.2.
https://www.ncbi.nlm.nih.gov/books/NBK554919/

10. NICE. Suspected neurological conditions recognition and referral. NG127. 2019; updated 2023. Recommendation 1.7.3 and limb weakness recommendations.
https://www.ncbi.nlm.nih.gov/books/NBK616002/

11. NICE. Abdominal aortic aneurysm diagnosis and management. NG156. 2020. Recommendations 1.1.4 and 1.1.7–1.1.9.
https://www.ncbi.nlm.nih.gov/books/NBK556921/

12. Berbari EF et al. IDSA Clinical Practice Guidelines for the Diagnosis and Treatment of Native Vertebral Osteomyelitis in Adults. 2015. Recommendations 1, 3, 5–8, 13, 17 and 25; diagnostic evidence summary.
https://doi.org/10.1093/cid/civ482

13. Finucane LM et al. International Framework for Red Flags for Potential Serious Spinal Pathologies. J Orthop Sports Phys Ther. 2020;50:350–372. Relevant sections: clinical reasoning, malignancy and fracture.
https://doi.org/10.2519/jospt.2020.9971

14. Delitto A et al. Low Back Pain Clinical Practice Guidelines. J Orthop Sports Phys Ther. 2012;42:A1–A57. Relevant section: differential diagnosis, including failure to improve within 30 days.
https://doi.org/10.2519/jospt.2012.42.4.A1

15. NICE. Spinal metastases and metastatic spinal cord compression. NG234. 2023. Box 1 and recommendations 1.3.1–1.3.5.
https://www.nice.org.uk/guidance/ng234/chapter/recommendations

16. NICE. Spondyloarthritis in over 16s diagnosis and management. NG65. 2017. Recommendation 1.1.5.
https://www.ncbi.nlm.nih.gov/books/NBK553609/
