# Supplementary material for: Clinical Decision Making, Red-Flag Recognition, and Scope-of-Practice Perspectives for Direct Access Physical Therapy in Saudi Arabia
Source: Healthcare (Basel). 2026 Oct 8;14(19):3347. doi: 10.3390/healthcare14193347 (PMC13664920; doi:10.3390/healthcare14193347)
Supplement: Supplementary file 1 [file healthcare-14-03347-s001.zip › File_S4_Consolidated_Analytical_Material.pdf]

Supplementary File S4

Clinical Decision-Making, Red-Flag Recognition, and Scope-of-Practice Perspectives for Direct Access Physical Therapy in Saudi Arabia

Consolidated Analytical Material

Cross-file note: Saudi nationals are the primary analytic cohort; eligible non-Saudi physical therapists form a secondary exploratory cohort. No overall readiness index, arbitrary threshold, or ready/not-ready classification is used. The complete questionnaire is reproduced in Supplementary File S5. Tables S7–S9 provide the questionnaire structure, contextual benchmark, and complete item-level red-flag results; Tables S10–S15 document diagnostics, sensitivity analyses, calibration, exploratory nationality comparisons, formal-training coding, and the V7 alternative-scoring sensitivity analysis. Sections S16–S19 document analytical chronology, measure definitions, revision-stage clinical and regulatory explanations, and post hoc sensitivity and response-distribution results. The submitted keys remain the baseline. Section S19 reports the additional post hoc scoring analyses; File S10 gives the item-level guideline-based appraisal, and File S11 provides the separate R code. Legacy R variable names and output labels are retained for reproducibility and do not establish clinical safety or independent key validity. Section S20 preserves additional administration and descriptive details; Supplementary Figure S1 displays the principal adjusted referral model. All five main figures and Supplementary Figure S1 are generated by the R script in S2.

SUPPLEMENTARY ANALYTICAL MATERIAL

Supplementary Section S1. Complete Questionnaire Grouping and Analytical Disposition

Table S1 lists the full question stems or item sets and identifies whether they contributed to a grouped outcome, a standalone analysis, eligibility or descriptive reporting, or qualitative analysis.

Table S1. Complete questionnaire grouping and analytical disposition.

Questionnaire section	Full question stem/items	Analytical disposition
Consent and eligibility	Q1 informed consent: confirmation that the participant had read and understood the Participant Information Sheet, was aged at least 18 years, participated voluntarily, and consented. Q2 professional eligibility: confirmation of ALL five criteria—current SCFHS registration; current clinical physical therapy practice in Saudi Arabia; at least one year of post-licensure experience; not an intern, student, or trainee; and not working exclusively in a non-clinical academic or administrative role.	Mandatory gates. No at Q1 or Q2 terminated the form. The export contained one non-consenting submission and 15 consented submissions that did not confirm all five professional criteria; no subsequent questionnaire data were present.
Demographic/professional characteristics	Age; sex; nationality; administrative region; primary practice sector; highest academic qualification; entry-level physical therapy degree; years of post-licensure clinical experience; primary clinical area; postgraduate certifications; medical-screening/differential-diagnosis training; prior direct-access practice.	Nationality cohort allocation, descriptive summaries, exploratory comparisons, and selected model predictors. Not combined into an outcome score.
Clinical vignettes	For each of 12 case presentations: What is the most appropriate management decision? Options: Treat without referral; Treat and concurrently consult/refer; Refer before physical therapy intervention.	Grouped into strict-key, graded-appropriateness, Jette-compatible, musculoskeletal, medically concerning, and safety-related urgent/critical referral outcomes. Full key in Table S2.

Questionnaire section	Full question stem/items	Analytical disposition
Red-flag awareness	For each of 20 warning features: Do you consider this a red flag warranting further investigation or referral? Responses: Yes; No; Not sure.	Grouped into total study-key agreement, core awareness, context-dependent keyed agreement, and seven domain summaries. Full key in Table S3.
Reported screening frequency	For each of the same 20 warning features: How frequently do you screen for this during the initial assessment? Responses: Never; Rarely; Sometimes; Often; Always.	Item/domain analyses and descriptive frequency sum 0-80. No correct answer and no readiness interpretation.
Red-flag confidence	Overall, how confident are you in identifying red flags during initial assessment?	Standalone 1–5 self-confidence outcome; correlated with standardised keyed scores.
Regulatory awareness	Are you aware of the current regulations governing direct access to physical therapy in Saudi Arabia?	Standalone self-rated awareness: 0=not aware, 1=partially aware, 2=fully aware.
Regulatory-status interpretation	In your understanding, is direct access to physical therapy currently permitted in Saudi Arabia?	Four-category response distribution plus study-specific binary agreement with the investigator interpretation of partial permission in limited settings.
Scope of practice	In your view, the legal scope of physical therapy practice in Saudi Arabia should include: initial assessment and diagnosis without referral; ordering plain radiographs; ordering MRI/CT; ordering laboratory investigations; direct referral to specialists; prescribing medications; therapeutic injections; discharge without physician sign-off; issuing sick-leave/fitness-for-work certificates.	Core, diagnostic, and extended-therapy grouped means. Administrative authority reported separately.
Stakeholder support	How supportive are the Saudi Physical Therapy Association, SCFHS, Ministry of Health policymakers, physicians, the Saudi public, medical insurance providers, and hospital/clinic administrators regarding direct access?	Seven-item support mean plus item-level distributions.
Educational preparation	Entry-level education adequately prepares graduates; postgraduate specialty training should be required; CPD requirements should be increased; a formal credentialing pathway is needed.	Entry-level adequacy analysed separately. The other three items form the additional-preparation-need mean.
Implementation barriers	Importance of: legal/regulatory framework, physician support, policymaker support, public awareness, insurance/reimbursement, entry-level education, postgraduate pathways, patient safety, medico-legal liability, evidence-based-practice culture, and cultural/institutional resistance.	All 11 items reported individually and combined as barrier-intensity sum 0-44.
Open-text recommendation	Please describe any specific training, support, or policy changes you feel are most important to enable direct-access practice in Saudi Arabia.	Descriptive coding using eight themes defined before reading the comments; multiple labels permitted. During revision, the author reviewed all 32 substantive comments against their assigned theme labels and corrected coding concerns with AI assistance. All 32 substantive comments form the denominator. No independent second coder was used. Supplementary File S8 provides translation notes, interpretations, and the coding audit.

Supplementary Section S2. Clinical Vignette Scoring Key

Table S2. Clinical vignette scoring key and analytical grouping.

The numerical strict and graded keys below are the submitted baseline. Graded 2/1/0 weights and study referral rules do not classify a respondent or every alternative intervention as safe or unsafe. Clinical support, exact-key uncertainty, and selected alternatives are described in S10; revised scenario results are confined to Section S19.

ID	Presentation summary	Class	Strict key	Graded score	Study referral rule	Jette-compatible credit
V1	Lateral elbow pain after gardening; local provocation; no neurological or systemic features.	MS	1	1=2; 2=1; 3=0	1 or 2 credited	1 or 2
V2	Anterior knee pain with stairs/prolonged sitting; no effusion, instability, locking, or systemic concern.	MS	1	1=2; 2=1; 3=0	1 or 2 credited	1 or 2
V3	Acute mechanical low-back pain after lifting; local symptoms; no radicular, systemic, or red-flag features.	MS	1	1=2; 2=1; 3=0	1 or 2 credited	1 or 2
V4	Progressive capsular-pattern shoulder stiffness; equal active/passive restriction; no trauma or systemic concern.	MS	1	1=2; 2=1; 3=0	1 or 2 credited	1 or 2
V5	Bilateral plantar heel pain with first-step pattern; local tenderness; no systemic or neurological features.	MS	1	1=2; 2=1; 3=0	1 or 2 credited	1 or 2
V6	Cervical radicular pain with positive Spurling; intact strength, reflexes, and sensation; no systemic/CES features.	MS	1	1=2; 2=1; 3=0	1 or 2 credited	1 or 2
V7	Long-standing diabetes with progressive bilateral foot paraesthesia and unsteadiness; no recent medical follow-up.	Medically concerning	2	2=2; 3=1; 1=0	2 or 3 credited	2 or 3
V8	Shoulder pain with fatigue and intermittent palpitations; unclear cardiac history.	Medically concerning	2 or 3	2/3=2; 1=0	2 or 3 credited	2 or 3
V9	Daily NSAID use with dark stools, fatigue, and mild tachycardia.	Urgent/critical	3	3=2; 1/2=0	3 only	3 only
V10	Severe low-back pain with urinary retention, saddle anaesthesia, bilateral weakness, and reduced perianal sensation.	Urgent/critical	3	3=2; 1/2=0	3 only	3 only
V11	Sudden severe throbbing back pain radiating to abdomen; pulsatile abdominal mass and vascular risk.	Urgent/critical	3	3=2; 1/2=0	3 only	3 only
V12	Intravenous drug use with severe back pain, fever, night sweats, rest/night pain, and rapid weight loss.	Urgent/critical	3	3=2; 1/2=0	3 only	3 only

Supplementary Section S3. Red-Flag Awareness Key and Clinical Domains

Table S3. Red-flag awareness answer key, scoring, and clinical-domain allocation.

ID	Full warning-feature item	Key	Domain	Awareness coding
RF1	Age >50 years with new-onset back pain	No	Context-dependent features	1 for the study-key response; 0 otherwise
RF2	Age <20 years with new-onset back pain	No	Context-dependent features	1 for the study-key response; 0 otherwise
RF3	Personal history of cancer/malignancy	Yes	Malignancy/systemic	1 for the study-key response; 0 otherwise
RF4	Unexplained weight loss >10% in 3-6 months	Yes	Malignancy/systemic	1 for the study-key response; 0 otherwise
RF5	Constant, progressive, non-mechanical pain	Yes	Malignancy/systemic	1 for the study-key response; 0 otherwise
RF6	Severe night pain unrelieved by position or rest	Yes	Malignancy/systemic	1 for the study-key response; 0 otherwise
RF7	Fever or recent bacterial infection	Yes	Infection	1 for the study-key response; 0 otherwise
RF8	Intravenous drug use	Yes	Infection	1 for the study-key response; 0 otherwise
RF9	Immunosuppression	Yes	Infection	1 for the study-key response; 0 otherwise
RF10	Significant trauma proportional to age and bone health	Yes	Fracture	1 for the study-key response; 0 otherwise
RF11	Saddle anaesthesia/perineal numbness	Yes	CES/neurological	1 for the study-key response; 0 otherwise
RF12	Recent bladder dysfunction	Yes	CES/neurological	1 for the study-key response; 0 otherwise
RF13	Recent bowel incontinence	Yes	CES/neurological	1 for the study-key response; 0 otherwise
RF14	Bilateral lower-limb weakness or sensory loss	Yes	CES/neurological	1 for the study-key response; 0 otherwise
RF15	Progressive neurological deficit	Yes	CES/neurological	1 for the study-key response; 0 otherwise
RF16	Severe morning stiffness >1 hour in a younger patient	Yes	Inflammatory	1 for the study-key response; 0 otherwise
RF17	Pulsatile abdominal mass	Yes	Vascular	1 for the study-key response; 0 otherwise

ID	Full warning-feature item	Key	Domain	Awareness coding
RF18	Severe non-mechanical throbbing back pain in an older person with cardiovascular risk	Yes	Vascular	1 for the study-key response; 0 otherwise
RF19	Prolonged corticosteroid use	Yes	Fracture	1 for the study-key response; 0 otherwise
RF20	Failure of pain to respond to any treatment over >4 weeks	No	Context-dependent features	1 for the study-key response; 0 otherwise

Supplementary Section S4. Derived Variables and Score Ranges

Table S4. Derived variables, ranges, and derivation rules.

Variable	Definition	Range	Derivation
DM_STRICT	Strict-key vignette count	0-12	1 for each study-defined optimal response; V8 accepts 2 or 3
DM_GRADED	Graded vignette appropriateness	0-24	Sum of 0/1/2 points across V1-V12
DM_GRADED_MS	Musculoskeletal graded score	0-12	V1-V6
DM_GRADED_MED	Medically concerning graded score	0-4	V7-V8
DM_GRADED_CRIT	Urgent/critical graded score	0-8	V9-V12
DM_JETTE_RULE	Jette-compatible decision score	0-12	V1-V6: option 1 or 2; V7-V8: option 2 or 3; V9-V12: option 3
CRIT_SAFE_N	Urgent/critical vignettes referred before intervention	0-4	Number of V9-V12 with option 3
CRIT_ALL_SAFE	All four urgent/critical vignettes referred before intervention	0/1	1 only when V9-V12 all equal option 3
RF_AWARE_TOTAL	Red-flag awareness total	0-20	Sum of keyed responses RF1-RF20
RF_AWARE_CORE	Core warning-feature awareness	0-17	RF3-RF19
RF_DISCRIM	Context-dependent keyed agreement	0-3	RF1, RF2, and RF20 answered No under the retained exploratory study key
RF_USE_TOTAL	Reported screening-frequency sum	0-80	Never=0 to Always=4; frequency only
RF_CONF	Red-flag confidence	1-5	Standalone item
LEGAL_AWARE	Self-rated legal awareness	0-2	Not aware/partially aware/fully aware
LEGAL_STATUS_ALIGN	Agreement with investigator-defined regulatory interpretation	0/1	1 for partial permission in limited settings; 0 otherwise
SCOPE_CORE	Core direct-access scope mean	1-5	Assessment/diagnosis, specialist referral, discharge without sign-off
SCOPE_DIAG	Diagnostic-investigation authority mean	1-5	Radiographs, MRI/CT, laboratory investigations
SCOPE_EXT	Extended therapeutic authority mean	1-5	Medication prescribing and therapeutic injections
SCOPE_ADMIN	Administrative authority	1-5	Sick-leave/fitness-for-work certificate item

Variable	Definition	Range	Derivation
SUPPORT_MEAN	Stakeholder support mean	1-5	Mean of seven stakeholder items
EDU_ADEQ	Entry-level education adequacy	1-5	Standalone item
PREP_NEED	Additional preparation need mean	1-5	Postgraduate training, increased CPD, credentialing
BARRIER_SUM	Barrier-intensity sum	0-44	Sum of 11 barrier items
CRIT_NONREFERRAL_FIRST_N	Urgent/critical vignettes not referred before intervention	0–4	4 minus CRIT_SAFE_N
SCREEN_TRAINING_ANY	Formal medical-screening training	0/1	1 for entry-level education, postgraduate/specialty training, or external CPD course/workshop; self-directed study alone = 0

Supplementary Section S5. Statistical Model and Comparison Specifications

Table S5. Statistical model, comparison, and multiplicity specifications.

Outcome/analysis	Method	Predictors/comparison	Effect estimate	Multiplicity treatment
Referral before intervention for all four urgent/critical vignettes	Multivariable logistic regression	Postgraduate qualification; centred age; sex; MSK/sports practice; formal screening training (entry-level education, postgraduate/specialty training, or external CPD course/workshop; self-directed study alone excluded); prior direct-access exposure	Adjusted OR with Wald 95% CI	Holm across six coefficients within this model; Firth sensitivity reported separately
Core scope endorsement	Linear regression with HC3 standard errors	Same six predictors	Adjusted mean difference with t-based 95% CI	Holm across six coefficients within this model
Core vs extended scope	Paired Wilcoxon signed-rank test	Within-participant paired domain means	Paired rank-biserial correlation	Single defined paired comparison
Saudi vs non-Saudi continuous outcomes	Welch t test	Nationality cohort	Mean difference, 95% CI, Hedges g	Combined with the binary nationality comparison in one 18-outcome Holm family
Saudi vs non-Saudi referral before intervention for all four urgent/critical vignettes	Fisher exact test	Nationality cohort	Saudi-vs-non-Saudi exact OR and 95% CI	Combined with the 17 continuous nationality comparisons in one 18-outcome Holm family
Awareness subgroup comparisons	Welch t tests	Experience ≥10 years; formal training as defined above; entry-level DPT; MSK/sports practice; direct-access exposure	Mean difference and 95% CI	Holm across five comparisons
Awareness and behavioural associations	Spearman correlation	Awareness, screening frequency, confidence, vignette performance, and urgent/critical referral scores	Spearman rho and unadjusted p-value	Exploratory correlations; effect size emphasized; p values unadjusted
Internal consistency and item-rest summaries	KR-20, raw Cronbach alpha, item-rest point-biserial	Awareness, stakeholder support, barriers, preparation, vignette items	Internal-consistency coefficient or item-rest correlation; not clinical key validity	Interpretation cautious; no alpha for heterogeneous vignettes
Software environment and reproducibility	R 4.6.1 on Linux; exact environment and package versions in Supplementary File S3	De-identified input dataset (S1); complete R script (S2); sessionInfo record (S3)	Regenerates analytical tables, numerical results, and source figures in one run	Not applicable

Outcome/analysis	Method	Predictors/comparison	Effect estimate	Multiplicity treatment
Adjusted-model diagnostics	Linearity check, VIF, Cook distance, AUC, Brier score, calibration by risk decile, Breusch–Pagan test; Firth logistic sensitivity	Six-predictor urgent-referral and core-scope models	Diagnostic statistics and bias-reduced ORs with profile-likelihood 95% CIs	Diagnostic/sensitivity analyses; no separate multiplicity family
V7 alternative strict-scoring sensitivity	Post hoc recomputation of the strict-key score with V7 Options 2 or 3 credited	Primary V7 key (Option 2 only) versus alternative V7 key (Options 2 or 3)	Mean strict-key score and percent of maximum	Descriptive post hoc sensitivity analysis; no separate multiplicity family

Supplementary Section S6. Exploratory Red-Flag Awareness Subgroup Comparisons

Table S6. Exploratory red-flag awareness subgroup comparisons in the Saudi cohort (n = 131).

Comparison	Group 1 vs Group 0, n; mean (SD)	Mean difference (95% CI)	p; Holm p	Interpretation
Experience ≥10 years vs <10 years	50; 13.74 (3.99) vs 81; 14.06 (3.84)	−0.32 (−1.73 to 1.08)	0.650; 0.650	Not significant
Any formal screening training vs none	106; 14.26 (3.47) vs 25; 12.56 (5.16)	1.70 (−0.52 to 3.93)	0.128; 0.510	Not significant
Entry-level DPT vs Bachelor	8; 16.63 (1.30) vs 123; 13.76 (3.94)	2.86 (1.64 to 4.09)	0.00012; 0.00060	Significant after correction; exploratory
MSK/sports practice vs other practice	64; 14.30 (3.46) vs 67; 13.60 (4.26)	0.70 (−0.64 to 2.04)	0.303; 0.620	Not significant
Prior direct-access exposure vs none	88; 14.24 (3.90) vs 43; 13.33 (3.83)	0.91 (−0.51 to 2.34)	0.207; 0.620	Not significant

Group 1 minus Group 0 mean differences were estimated with Welch t tests. Holm adjustment was applied across the five exploratory comparisons. Total red-flag awareness ranged from 0 to 20. Formal screening training comprised entry-level education, postgraduate/specialty training, or an external continuing professional development course/workshop; self-directed study alone was excluded. DPT = Doctor of Physical Therapy; MSK = musculoskeletal.

Supplementary Section S7. Concise Questionnaire Structure and Principal Analysis

Table S7 summarises each survey block, item count, grouped outcome or range, and analytical treatment; Table S1 provides the more detailed question-by-question disposition.

Table S7. Questionnaire structure, question grouping, and principal analysis.

Survey block	Items	Grouped outcome/range	How analysed
Eligibility and background	2 gates + 12 background variables	No outcome score	Consent and professional eligibility filters; nationality cohort allocation; descriptive variables; selected model predictors
Clinical management vignettes	12 vignettes	Strict-key 0–12; graded 0–24; Jette-compatible 0–12; urgent/critical referral 0–4	Overall, item-level, and three clinical-category analyses
Red-flag awareness	20 items	Total 0-20; core 0-17; context-dependent agreement 0-3; seven domain summaries	Study-key agreement with Wilson confidence intervals; raw responses also reported
Reported screening frequency	20 matched items	Item/domain ratings; descriptive sum 0-80	Frequency only; not interpreted as correctness or readiness
Red-flag confidence	1 item	1-5	Standalone self-perception and correlation analysis
Regulatory awareness and legal status	2 items	Awareness 0–2; agreement with the investigator interpretation 0/1	Four-category regulatory distribution and separate investigator-interpretation agreement summary
Scope endorsement	9 items	Core, diagnostic, and extended-therapy means 1-5; administrative item separate	Defined construct-specific domains
Stakeholder support	7 items	Mean 1-5 plus item distributions	Grouped only when internal consistency was acceptable
Educational preparation	4 items	Entry-level adequacy separate; additional-preparation mean 1-5	Three-item preparation-need scale; adequacy remains standalone
Implementation barriers	11 items	Item ratings 0-4; barrier sum 0-44	Item ranking and overall barrier intensity
Open-text recommendations	1 optional question	No numeric score	Descriptive content coding using eight predefined themes; labels reviewed by the author during revision, with multiple labels allowed; all 32 substantive comments classified. Frequencies describe aspects mentioned. No independent second coder. See Supplementary File S8.

Supplementary Section S8. Jette-Compatible Contextual Benchmark

Table S8 provides a three-category contextual comparison of this study's Jette-compatible percentages with the rounded values reported by Jette et al.; the comparison is descriptive and does not imply instrument equivalence.

Table S8. Contextual comparison using Jette-compatible decision rules.

Condition category	This study, %	Jette et al., %	Difference, pp
Musculoskeletal	88.2	87	+1.2
Medically concerning / non-critical	91.6	88	+3.6
Urgent / critical	76.5	79	−2.5

Supplementary Section S9. Item-Level Red-Flag Awareness and Screening

Table S9 reports study-key agreement, Yes responses, frequent screening, and the descriptive recognition–screening difference. Table S18b additionally reports Yes, No, and Not sure separately for every item. The original question combined further investigation or referral; the three negatively keyed items do not establish independent-referral discrimination or inappropriate overcalling.

Table S9. Item-level red-flag awareness and reported screening frequency (Saudi cohort, n = 131).

Warning feature	Key	Study-key %	Rated Yes %	Screened often/always %	Difference, pp
RF1 Age > 50 years with new onset of back pain	No	56.5	35.9	65.6	—
RF2 Age < 20 years with new onset of back pain	No	62.6	26.0	51.1	—
RF3 History of cancer	Yes	88.5	88.5	52.7	+35.9
RF4 Unexplained weight loss	Yes	87.8	87.8	51.1	+36.6
RF5 Constant progressive pain	Yes	66.4	66.4	52.7	+13.7
RF6 Severe unrelieved night pain	Yes	77.9	77.9	55.0	+22.9
RF7 Fever / recent infection	Yes	75.6	75.6	46.6	+29.0
**RF8 Intravenous drug use**	**Yes**	**54.2**	**54.2**	**40.5**	**+13.7**
RF9 Immunosuppression	Yes	67.2	67.2	39.7	+27.5
RF10 Significant trauma	Yes	70.2	70.2	51.1	+19.1
RF11 Saddle anaesthesia	Yes	79.4	79.4	49.6	+29.8

Warning feature	Key	Study-key %	Rated Yes %	Screened often/always %	Difference, pp
RF12 Recent bladder dysfunction	Yes	83.2	83.2	51.1	+32.1
RF13 Recent bowel incontinence	Yes	83.2	83.2	51.9	+31.3
RF14 Bilateral lower-limb weakness	Yes	80.9	80.9	56.5	+24.4
RF15 Progressive neurological deficit	Yes	75.6	75.6	53.4	+22.1
RF16 Morning stiffness >1 h	Yes	54.2	54.2	53.4	+0.8
RF17 Pulsatile abdominal mass	Yes	78.6	78.6	39.7	+38.9
RF18 Non-mechanical throbbing pain	Yes	78.6	78.6	50.4	+28.2
**RF19 Prolonged corticosteroid use**	**Yes**	**55.7**	**55.7**	**39.7**	**+16.0**
**RF20 Failure of pain to respond to any treatment over > 4 weeks**	**No**	**17.6**	**66.4**	**45.8**	—

Supplementary Section S10. Adjusted-Model Diagnostics

Table S10 summarises diagnostics for the six-predictor logistic model of referral before intervention for all four urgent/critical vignettes and the six-predictor core-scope linear model. The diagnostics assess model form, multicollinearity, influence, discrimination, calibration, and heteroscedasticity; they do not establish external predictive validity.

Table S10. Adjusted-model diagnostics (Saudi cohort, n = 131).

Model	Diagnostic	Result
Referral-before-intervention logistic model (all four urgent/critical vignettes)	Outcome counts	77 events; 54 non-events
Referral-before-intervention logistic model (all four urgent/critical vignettes)	Age linearity in the logit	Age × log(age) p = 0.360
Referral-before-intervention logistic model (all four urgent/critical vignettes)	Multicollinearity	Maximum VIF = 1.34
Referral-before-intervention logistic model (all four urgent/critical vignettes)	Apparent discrimination	AUC = 0.685
Referral-before-intervention logistic model (all four urgent/critical vignettes)	Overall prediction error	Brier score = 0.216
Referral-before-intervention logistic model (all four urgent/critical vignettes)	Influence	Maximum Cook distance = 0.071; 3 observations > 4/n
Core-scope linear model	Multicollinearity	Maximum VIF = 1.34
Core-scope linear model	Heteroscedasticity	Breusch–Pagan p = 0.804

Model	Diagnostic	Result
Core-scope linear model	Influence	Maximum Cook distance = 0.090; 7 observations > 4/n
Referral-before-intervention logistic model (all four urgent/critical vignettes)	Calibration by predicted-risk decile	Variable across groups of 13–14 participants; detailed results in Table S12

Supplementary Section S11. Firth Bias-Reduced Logistic Sensitivity Analysis

Table S11 reports a Firth bias-reduced sensitivity model using the same six predictors as the maximum-likelihood logistic model. Profile-likelihood confidence intervals are shown. The sensitivity analysis produced similar effect directions and magnitudes for postgraduate qualification and age.

Table S11. Firth bias-reduced logistic sensitivity analysis for referral before intervention for all four urgent/critical vignettes (Saudi cohort, n = 131).

Predictor	OR	95% CI	Profile-likelihood p
Postgraduate academic qualification	3.78	1.59–9.77	0.002
Age, per additional year	0.92	0.86–0.98	0.008
Female sex	1.47	0.69–3.18	0.317
MSK/sports practice	0.74	0.34–1.56	0.427
Any formal screening training	0.80	0.31–2.02	0.644
Any prior direct-access exposure	0.83	0.38–1.80	0.637

Supplementary Section S12. Logistic-Model Calibration

Table S12 presents descriptive calibration by predicted-risk decile for the six-predictor logistic model of referral before intervention for all four urgent/critical vignettes. Each group contains only 13–14 participants; the values are therefore descriptive and do not establish external predictive validity.

Table S12. Calibration by predicted-risk decile for referral before intervention for all four urgent/critical vignettes (Saudi cohort, n = 131).

Risk decile	n	Mean predicted probability	Observed proportion
1	14	0.274	0.214
2	13	0.423	0.538
3	13	0.485	0.538
4	13	0.544	0.538
5	13	0.597	0.538
6	13	0.624	0.692
7	13	0.652	0.769
8	13	0.706	0.308
9	13	0.762	0.769
10	13	0.835	1.000

Supplementary Section S13. Exploratory Nationality Comparisons

Tables S13a and S13b report the complete exploratory Saudi versus non-Saudi comparisons. The 17 continuous outcomes and the binary referral-before-intervention outcome formed one 18-outcome Holm family. The non-Saudi cohort contained 12 participants; results are descriptive and do not establish equivalence.

Table S13a. Continuous Saudi versus non-Saudi comparisons (18-outcome Holm family).

Outcome	Saudi mean (SD)	Non-Saudi mean (SD)	Mean difference (95% CI)	Hedges g	Raw p	Holm p
Strict-key vignette score (0–12)	7.83 (2.48)	7.67 (2.61)	0.17 (−1.53 to 1.86)	0.07	0.836	1.000
Graded vignette score (0–24)	18.02 (4.25)	17.75 (4.43)	0.27 (−2.62 to 3.15)	0.06	0.845	1.000
Jette-compatible score (0–12)	10.18 (2.06)	10.08 (2.11)	0.10 (−1.27 to 1.47)	0.05	0.877	1.000
Urgent/critical vignettes referred first (0–4)	3.06 (1.42)	2.83 (1.59)	0.23 (−0.80 to 1.26)	0.16	0.639	1.000
Total red-flag awareness (0–20)	13.94 (3.89)	14.25 (4.86)	−0.31 (−3.45 to 2.83)	−0.08	0.833	1.000

Outcome	Saudi mean (SD)	Non-Saudi mean (SD)	Mean difference (95% CI)	Hedges g	Raw p	Holm p
Core warning-feature awareness (0–17)	12.57 (3.96)	12.92 (4.60)	−0.34 (−3.32 to 2.63)	−0.09	0.806	1.000
Context-dependent keyed agreement (0–3)	1.37 (0.90)	1.33 (0.89)	0.03 (−0.55 to 0.61)	0.04	0.904	1.000
Screening-frequency sum (0–80)	47.31 (24.94)	58.42 (21.19)	−11.10 (−25.03 to 2.83)	−0.45	0.109	1.000
Red-flag confidence (1–5)	3.76 (0.86)	4.42 (0.67)	−0.65 (−1.10 to −0.21)	−0.77	0.007	0.121
Self-rated regulatory awareness (0–2)	1.21 (0.68)	1.58 (0.79)	−0.38 (−0.89 to 0.14)	−0.55	0.136	1.000
Core scope endorsement (1–5)	3.74 (1.11)	4.28 (0.74)	−0.53 (−1.03 to −0.04)	−0.49	0.036	0.505
Diagnostic/investigation endorsement (1–5)	3.60 (1.17)	4.06 (0.83)	−0.46 (−1.01 to 0.10)	−0.39	0.100	1.000
Extended therapeutic endorsement (1–5)	3.06 (1.27)	3.92 (0.90)	−0.86 (−1.46 to −0.26)	−0.69	0.008	0.137
Stakeholder support mean (1–5)	3.32 (1.01)	3.80 (0.75)	−0.48 (−0.98 to 0.02)	−0.48	0.059	0.770
Entry-level education adequacy (1–5)	2.93 (1.30)	3.67 (0.89)	−0.74 (−1.33 to −0.14)	−0.57	0.019	0.280
Additional preparation need (1–5)	3.90 (1.12)	4.33 (0.45)	−0.43 (−0.77 to −0.10)	−0.40	0.013	0.209
Barrier-intensity sum (0–44)	25.36 (9.71)	28.58 (7.69)	−3.22 (−8.31 to 1.86)	−0.34	0.196	1.000

Table S13b. Referral before intervention for all four urgent/critical vignettes by nationality (18-outcome Holm family).

Outcome	Saudi n/N (%)	Non-Saudi n/N (%)	OR (Saudi vs non-Saudi)	Exact 95% CI	Raw Fisher p	Holm p
Referral before intervention for all four urgent/critical vignettes	77/131 (58.8%)	6/12 (50.0%)	1.42	0.36 to 5.63	0.559	1.000

Supplementary Section S14. Formal Screening-Training Coding Audit

Table S14 documents the author-approved formal-source definition used in all reported models. Formal training includes entry-level education, postgraduate/specialty training, or an external CPD course/workshop; self-directed study alone is not classified as formal training.

Table S14. Formal screening-training classification audit (Saudi cohort, n = 131).

Audit item	n
Primary formal-source coding: formal training = 1	106
Primary formal-source coding: no formal training = 0	25
Broader historical coding: any learning source except explicit no formal training = 1	114
Broader historical coding: no formal training = 0	17
Records changed by formal-source definition	8
Self-directed study only	8
Self-directed study plus 'No formal training'	2

Supplementary Section S15. V7 Alternative Strict-Scoring Sensitivity Analysis

Table S15 reports the original post hoc V7 analysis accepting either Option 2 (Treat and Consult/Refer) or Option 3 (Refer First). The submitted V7 key (Option 2 only) remains the baseline; prospective specification is not established. Additional V7/V8 and warning-feature scoring analyses and their association results appear in Section S19, with clinical rationale in S10 and separate R code in S11.

Table S15. Post hoc V7 alternative strict-scoring sensitivity analysis (Saudi cohort, n = 131).

Scoring rule	Mean strict score (0–12)	SD	Median	Percent of maximum
Baseline: V7 Option 2 only	7.83	2.48	8	65.3%
Post hoc sensitivity: V7 Option 2 or 3	8.46	2.53	9	70.5%

The 17 core items were all positively keyed, so the score may partly reflect a general tendency to endorse Yes. Internal-consistency coefficients and item-rest correlations do not establish clinical correctness of the answer keys, content validity, or broader questionnaire reliability. The regulatory binary variable is an investigator-defined interpretation, not an authoritative test of national legal knowledge.

Supplementary Section S16 Analytical chronology and interpretation

Table S16a. Documented analytical decisions and limitations of timing evidence.

Decision	Available record	Interpretation
Four-vignette primary referral outcome	Post-collection analysis-plan revisions documented the four-vignette composite definition and safety-focused outcomes.	The date when this became the single primary outcome cannot be established. It is the primary outcome for this report; prospective specification is not claimed.
Vignette and warning-feature keys	Earlier and final keys differed; post-collection analysis-plan revisions documented revised classifications and the final 20-item framework.	The final keys cannot be verified as precollection specifications. The submitted keys remain the baseline; revision-stage appraisal and selected alternative-key analyses are documented in S10 and Section S19.
Saudi-national primary cohort	Saudi workforce policy was the primary focus; eligibility included non-Saudis. The explicit Saudi-primary analysis plan was documented after collection.	An earlier date of the analytical restriction cannot be established. All-eligible descriptive sensitivities are now provided.
Six adjusted-model covariates	Earlier records support clinical relevance, parsimony, sparse categories, and overlap as reasons for a limited model.	The exact timing relative to initial outcome-association inspection cannot be verified; adjusted analyses are exploratory.
Holm comparison families	Holm adjustment appeared in earlier analyses; final comparison families were refined in the analysis scripts, including one 18-outcome nationality family.	Precollection specification of the final families is not established.
Revision analyses and explanations	Expanded and age–experience models, pooled descriptions, and literature-linked referral and regulatory explanations were developed during revision.	These additions are post hoc; they do not retrospectively establish the original decision sequence or independent validation.

The manually performed demographic review compared age with years of post-licensure experience and highest academic qualification. The author identified no inconsistency warranting exclusion during that review, and no participant was excluded on this basis. This is distinct from independent verification. Entry-level degree and highest qualification were separate responses: eight Saudi respondents reported entry-level DPT, whereas highest qualification included seven DPT and two Other responses. One entry-level DPT response paired with Bachelor’s highest qualification remains unresolved and was retained as reported.

Supplementary Section S16 continued Measure definitions

Table S16b. Principal measures and response coding.

Measure	Components	Responses	Scoring and direction	Range
Four-vignette referral composite	V9–V12	1 Treat without referral; 2 Treat and concurrently consult/refer; 3 Refer before any PT	1 only when Option 3 is selected for every vignette; 0 otherwise	0–1
Strict-key vignette score	V1–V12	Three management options	Sum of study-key matches; higher means greater agreement with the study-defined optimum	0–12
Graded vignette score	V1–V12	Three management options	Each response receives study-defined 0/1/2; higher indicates greater graded agreement	0–24
Core warning-feature recognition	RF3–RF19	Yes; No; Not sure	Yes=1, No/Not sure=0; higher indicates more core features endorsed	0–17
Context-dependent keyed agreement	RF1, RF2, RF20	Yes; No; Not sure	No=1, Yes/Not sure=0 under the retained exploratory key; interpret with raw distributions	0–3
All-item awareness score	RF1–RF20	Yes; No; Not sure	Sum of retained study-key matches; exploratory	0–20
Screening-frequency sum	RF1–RF20 utilization	Never/Rarely/Sometimes/Often/Always	Responses coded 0/1/2/3/4 and summed; higher reported frequency	0–80
Core frequent-screening estimate	RF3–RF19 utilization	Never/Rarely/Sometimes/Often/Always	Item-level mean percentage choosing Often/Always; not a participant classifier	0–100%
Core scope endorsement	Scope items 1,5,8	Strongly disagree to strongly agree	Mean of responses coded 1–5; higher endorsement	1–5
Stakeholder support	Seven stakeholder items	Strongly opposed to strongly supportive	Mean of responses coded 1–5; higher perceived support	1–5
Preparation need	Education items 2–4	Strongly disagree to strongly agree	Mean of responses coded 1–5; higher additional preparation need	1–5
Perceived barriers	Eleven barrier items	Not a barrier/Minor/Moderate/Major/Critical	Sum coded 0–4; higher perceived barriers	0–44

Other scope means are defined in Table S4: diagnostic-investigation authority uses three items, extended therapeutic authority two, and administrative authority one. All use 1–5 endorsement responses. The 1–5 confidence item and 0–2 self-rated regulatory awareness item are separate single-item measures. No grand readiness total is calculated.

Supplementary Section S16 continued Clinical referral rationale

This investigator-prepared literature synthesis was developed during revision, with AI assistance disclosed in the manuscript. It is not independent validation. Table S16c explains V9–V12; File S10 appraises all items, including V7/V8 uncertainty.

The composite required referral before physiotherapy for each of four urgent/critical vignettes. Table S16c links clinical concern, urgency, and destination to literature. These abbreviated hypothetical presentations neither establish diagnoses nor imply identical acuity.

Table S16c. Literature-linked rationale for the retained V9–V12 referral-first rule.

Vignette and features	Clinical concern	Urgency and destination	Accepted response and source
V9: possible gastrointestinal bleeding<br>Daily ibuprofen use, dark stools, increasing fatigue, and mild tachycardia.	This combination raises concern for gastrointestinal bleeding and possible blood loss, requiring medical assessment rather than routine knee treatment alone.	Same-day acute medical assessment, usually through an emergency department or acute medical service with access to gastrointestinal evaluation; immediate emergency escalation if unstable or actively bleeding.	Option 3: Refer First<br>NICE_CG141; 1.1.1; 1.3.1–1.3.2
V10: suspected cauda equina syndrome<br>New urinary retention, saddle sensory loss, bilateral weakness, and reduced perianal sensation with severe low-back pain.	The new bladder and perineal neurological findings require immediate assessment for cauda equina compression.	Immediate emergency referral through the local cauda equina/spinal emergency pathway, with access to urgent MRI and spinal surgical assessment.	Option 3: Refer First<br>NICE_NG127; 1.7.3
V11: suspected symptomatic abdominal aortic aneurysm<br>Sudden severe non-mechanical back pain radiating to the abdomen, a pulsatile abdominal mass, older age, and vascular risk factors.	The presentation raises concern for symptomatic or ruptured abdominal aortic aneurysm; the recorded blood pressure of 165/95 mmHg does not exclude an aortic emergency.	Immediate emergency transfer for aortic assessment and vascular surgical input; treatment should not await a routine outpatient appointment.	Option 3: Refer First<br>NICE_NG156; 1.1.7–1.1.9
V12: suspected spinal infection<br>Severe persistent back pain, fever, night sweats, weight loss, and intravenous drug use.	New or worsening back pain with fever warrants investigation for spinal infection; intravenous drug use adds risk.	Urgent same-day acute medical assessment with access to blood tests, cultures, and spinal MRI, with infection/spine specialist input; immediate emergency escalation for sepsis, neurological compromise, or haemodynamic instability.	Option 3: Refer First<br>IDSA_NVO2015;<br>Recommendations 1, 6–8, 13, 17

Same-day referral for V9 and V12 is the investigator's precautionary interpretation, not a literal NICE or IDSA answer key. Guidelines distinguish unstable or neurologically compromised patients from stable presentations. None of the vignette diagnoses is confirmed.

Concurrent treatment and referral does not mean no referral was intended. Its clinical acceptability depends on intervention, stability, and referral timing, which were not recorded. Of the 54 participants not meeting the composite, 26 selected referral first in three vignettes, five in two, five in one, and 18 in none. Among all 131 participants, 36 (27.5%) selected concurrent treatment and referral in every non-referral-first vignette and never treatment without referral; 18 (13.7%) selected treatment without referral at least once, including nine for all four.

Supplementary Section S16 continued Regulatory documentation

All four regulatory responses were reported descriptively. The binary summary represents agreement with the investigator's interpretation of partial permission in limited settings, not verified national regulatory knowledge. MOH Annex 35 (version 4, section Alef, PDF p. 1; section Kha, PDF p. 17) permits independent physiotherapy centres and describes musculoskeletal diagnosis and treatment within their scope, but does not explicitly authorize patient self-referral. Annex 31 (version 4, section 2(a), PDF p. 2) describes physician-requested home physiotherapy services and supporting medical-report requirements for specified categories of home treatment. MOH public guidance, updated 20 October 2025, describes a referral and medical-report pathway for government hospitals. These documents concern different settings and do not establish a single nationwide self-referral standard. Publication/effective dates were not stated in the retrieved annex PDFs; their applicability throughout collection could not therefore be independently dated from those documents.

The parent Private Health Institutions Law and Its Implementing Regulations, fourth edition (2023), was also checked during revision. Article 1(8)(a) includes physiotherapy among auxiliary health-service centres. Implementing provisions under Article 15, item 4, refer home-care centres to Annex 31 and physiotherapy centres to Annex 35. This identifies the licensing framework and annex applicability but does not establish explicit patient self-referral permission. The edition year of the parent volume does not independently date the separately retrieved annex PDFs.

Table S16d. Setting-specific official documents and limits of interpretation.

Document	Provision and setting	Version and dates	Interpretive limit
Private Health Institutions Law and Its Implementing Regulations	Article 1(8)(a), PDF p. 3: physiotherapy as an auxiliary health-service centre. Implementing Article 15, item 4, PDF p. 20: Annexes 31 and 35 apply to home care and physiotherapy centres.	Fourth edition, 2023; checked during revision on 26 September 2026.	Licensing framework and annex applicability; no explicit patient self-referral permission.
Annex 35: Physical Therapy Centres; version 4	Section Alef, PDF p. 1: affiliated or independent centre. Section Kha, PDF p. 17: independent/affiliated musculoskeletal service, diagnosis and treatment.	No publication/effective date visible in the PDF; retrieved 26 September 2026.	Licensing and scope provisions. Not explicit patient self-referral authorization.
Annex 31: Home Medical Care Centres; version 4	Section 2(a), PDF p. 2: home physiotherapy scope includes physician-requested services; specified categories require a supporting medical report recommending dedicated physiotherapy.	No publication/effective date visible in the PDF; retrieved 26 September 2026.	Setting-specific physician/report provisions. Do not expand into a universal requirement for all listed routine services.
Physical Therapy; MOH public information	Section 'How to access medical rehabilitation and physical therapy services in government hospitals': referral via primary-care/hospital outpatient clinics, current medical report, registration and appointment.	Article last update: 20 October 2025. Site-wide footer last-modified date is different and should not be used as the article date.	Government-hospital public pathway guidance, not legislation proving or disproving self-referral throughout the private sector.

Sources for Tables S16c–S16d

MOH parent regulation: Ministry of Health, Kingdom of Saudi Arabia. Private Health Institutions Law and Its Implementing Regulations, 4th ed.; 2023. Available online: https://www.moh.gov.sa/Ministry/Rules/Documents/Private-Health-Institutions-Law.pdf (accessed on 26 September 2026).

NICE_CG141: National Institute for Health and Care Excellence. Acute Upper Gastrointestinal Bleeding in Over 16s: Management. Clinical Guideline CG141; published 13 June 2012; updated 25 August 2016. Available online: https://www.ncbi.nlm.nih.gov/books/NBK554919/ (accessed on 6 October 2026).

NICE_NG127: National Institute for Health and Care Excellence. Suspected Neurological Conditions: Recognition and Referral. NICE Guideline NG127; published 1 May 2019; updated 2 October 2023. Available online: https://www.ncbi.nlm.nih.gov/books/NBK616002/ (accessed on 6 October 2026).

NICE_NG156: National Institute for Health and Care Excellence. Abdominal Aortic Aneurysm: Diagnosis and Management. NICE Guideline NG156; published 19 March 2020. Available online: https://www.ncbi.nlm.nih.gov/books/NBK556921/ (accessed on 6 October 2026).

IDSA_NVO2015: Berbari, E.F.; Kanj, S.S.; Kowalski, T.J.; Darouiche, R.O.; Widmer, A.F.; Schmitt, S.K.; Hendershot, E.F.; Holtom, P.D.; Huddleston, P.M., III; Petermann, G.W.; Osmon, D.R. 2015 Infectious Diseases Society of America Clinical Practice Guidelines for the Diagnosis and Treatment of Native Vertebral Osteomyelitis in Adults. Clin. Infect. Dis. 2015, 61, e26–e46. https://doi.org/10.1093/cid/civ482.

MOH_Annex35: Ministry of Health, Kingdom of Saudi Arabia. Annex 35: Physical Therapy Centres, Version 4; undated. Available online: https://www.moh.gov.sa/eServices/Licences/Documents/35.pdf (accessed on 26 September 2026).

MOH_Annex31: Ministry of Health, Kingdom of Saudi Arabia. Annex 31: Home Medical Care Centres, Version 4; undated. Available online: https://www.moh.gov.sa/eServices/Licences/Documents/0031.pdf (accessed on 26 September 2026).

MOH_PTGuide: Ministry of Health, Kingdom of Saudi Arabia. Physical Therapy; updated 20 October 2025. Available online: https://www.moh.gov.sa/en/awarenessplateform/varioustopics/pages/physicaltherapy.aspx (accessed on 26 September 2026).

Supplementary Section S17 Post hoc covariate sensitivity analyses

Twelve background domains were recorded. All 11 varying Saudi domains had recorded responses; nationality was constant. During reconciliation with the original export, 59 literal None certification responses (55 Saudi) incorrectly converted to missing were restored. The Saudi counts were 55 None only, 72 named certification(s) without None, and four named certification(s) plus None (all 143 eligible respondents: 59, 80, and four, respectively); none was missing. Contradictory selections were retained without excluding participants. The indicators describe selected options, not adjudicated credential status. Region (12 categories) and certification types were omitted from the main model because of sparse categories and additional coefficients. Six certification-type indicators would add six coefficients, increasing the main model from six to 12. Sector also had numerous sparse categories (11); entry-level DPT comprised eight Saudi respondents. Full counts and coding are in S9.

Age was recorded numerically, whereas experience was recorded in six ordered categories. Their Spearman correlation was 0.894. Age uses one coefficient; entering experience as a categorical factor uses five without assuming an ordinal linear trend. This supports retaining age for parsimony, not its clinical superiority or a verified historical selection sequence. The main model uses six coefficients for 77 participants meeting the composite and 54 not meeting it: nine in the smaller category per coefficient. This is a limited-information association analysis, not a validated prediction model.

All model comparisons below use mean bias reduction (brglm2, Firth-type adjusted scores) with nominal asymptotic Wald 95% confidence intervals. Conventional logistic regression showed separation in the expanded 10-, 11-, 12-domain and fully detailed specifications; it did not show separation in the original or focused experience models. Bias reduction permits finite estimation but does not restore information lost through sparse cells or resolve confounding. These Wald intervals are distinct from the profile-likelihood intervals of the original logistf sensitivity in Table S11.

Table S17a. Bias-reduced logistic model comparisons for the four-vignette composite.

Model	n	Slope coefficients	Postgraduate OR (95% CI)	Age/year OR (95% CI)	ML separation
Submitted six-domain comparator	131	6	3.78 (1.51–9.43)	0.92 (0.86–0.98)	No
Age plus experience	131	11	3.33 (1.29–8.58)	0.85 (0.73–0.98)	No
Experience replaces age	131	10	2.69 (1.10–6.60)	Not included	No
Ten Saudi domains excluding certifications	131	33	4.27 (1.35–13.46)	0.91 (0.77–1.07)	Yes
All 11 varying Saudi domains	131	40	3.68 (0.97–13.97)	0.90 (0.75–1.08)	Yes
All 12 domains, pooled eligible	143	41	3.69 (1.07–12.73)	0.90 (0.78–1.04)	Yes
All original categories, Saudi	131	56	Detailed categories	0.98 (0.81–1.19)	Yes

The original six binary/numeric codings are retained in the 10-, 11-, and 12-domain expansions. Expanded certification coding includes six named-type indicators and separate None selection, preserving the four contradictions; removing the obsolete missingness indicator reduces affected models by one coefficient. The fully detailed 56-coefficient specification uses original qualification, specialty, training, exposure, and certification categories. This is a stress test, not a proposed main model, and greater apparent discrimination does not establish predictive validity. S9 provides coefficients, domain tests, inventories, and diagnostics.

Supplementary Section S17 continued Experience sensitivity

Table S17b. Focused experience-model estimates using mean bias reduction.

Model	Contrast	OR (95% CI)	Raw p	Holm p
Age plus experience	Postgraduate qualification	3.33 (1.29–8.58)	0.013	0.139
Age plus experience	Age, per year	0.85 (0.73–0.98)	0.026	0.264
Age plus experience	Experience: 3 to <6 years	2.33 (0.66–8.19)	0.187	0.992
Age plus experience	Experience: 6 to <10 years	4.36 (1.06–17.91)	0.041	0.368
Age plus experience	Experience: 10 to <15 years	5.15 (0.86–31.07)	0.074	0.589
Age plus experience	Experience: 15 to <20 years	5.76 (0.51–65.62)	0.158	0.992
Age plus experience	Experience: 20 years or more	10.70 (0.45–252.47)	0.142	0.992
Experience replaces age	Postgraduate qualification	2.69 (1.10–6.60)	0.031	0.306
Experience replaces age	Experience: 3 to <6 years	1.54 (0.47–4.99)	0.475	1.000
Experience replaces age	Experience: 6 to <10 years	1.93 (0.60–6.24)	0.274	1.000
Experience replaces age	Experience: 10 to <15 years	1.25 (0.35–4.45)	0.726	1.000
Experience replaces age	Experience: 15 to <20 years	0.69 (0.14–3.33)	0.648	1.000
Experience replaces age	Experience: 20 years or more	0.43 (0.09–1.95)	0.271	1.000

Experience reference category: 1 to <3 years. The remaining original covariates were sex, postgraduate qualification, MSK/sports practice, formal screening training, and prior direct-access exposure. In the model containing both age and experience, experience comparisons are conditional on age; strong overlap increases imprecision. Holm p values in Table S17b adjust all slope coefficients within that model, while the following block tests adjust across predictor domains.

Table S17c. Exploratory domain-level Wald tests in the focused experience models.

Model	Domain	df	Raw p	Holm p across domains
Age plus experience	Highest qualification	1	0.013	0.089
Age plus experience	Age	1	0.026	0.158
Age plus experience	Experience	5	0.468	1.000
Experience replaces age	Highest qualification	1	0.031	0.183
Experience replaces age	Experience	5	0.359	1.000

Expanded models yield wide intervals and model-dependent estimates. None of the domain associations in the expanded 10-, 11-, 12-domain or fully detailed specifications remained significant after their exploratory within-model Holm adjustments. The scope-endorsement expansions were also fitted; singleton categories generated leverage of one, making HC3 robust intervals unavailable for those expanded specifications. Such unavailable intervals are reported as unavailable in the R output, not replaced by purported robust estimates. Classical OLS output is provided separately for transparency and is not a substitute for the main HC3 analysis.

Supplementary Section S18 Pooled cohort and response distributions

Table S18a. Descriptive sensitivity including all eligible participants.

Measure	Saudi primary	Non-Saudi exploratory	All eligible
Participants	131	12	143
Referral first for all four, n (%)	77 (58.8)	6 (50.0)	83 (58.0)
Primary outcome Wilson 95% CI, %	50.2–66.8	25.4–74.6	49.8–65.8
Strict-key score, mean (SD)	7.83 (2.48)	7.67 (2.61)	7.82 (2.48)
Graded score, mean (SD)	18.02 (4.25)	17.75 (4.43)	17.99 (4.25)
Core warning-feature score, mean (SD)	12.57 (3.96)	12.92 (4.60)	12.60 (4.00)
All-item awareness, mean (SD)	13.94 (3.89)	14.25 (4.86)	13.97 (3.96)
Confidence, mean (SD)	3.76 (0.86)	4.42 (0.67)	3.82 (0.86)
Core scope endorsement, mean (SD)	3.74 (1.11)	4.28 (0.74)	3.79 (1.09)
Core Often/Always item mean, %	49.1	58.3	49.9
Investigator regulatory interpretation, n (%)	60 (45.8)	4 (33.3)	64 (44.8)

Exact Saudi screening calculation: 1,094 Often/Always responses across 2,227 participant–item observations (131 participants × 17 core features) = 49.1244%, reported as 49.1%. Core recognition was 1,647/2,227 = 73.9560%, reported as 74.0%. The difference calculated from the unrounded proportions was 24.8316 percentage points, reported as 24.8. This is the difference between descriptive item-level averages, not the percentage of participants who screened inadequately. The question concerned initial patient assessment and specified no recall period.

For complete responses, the mean of the 17 item-specific percentages equals the mean individual percentage across those same items. Neither calculation classifies participants as frequent screeners. Response-category detail follows; all percentages use n = 131 for each feature.

Supplementary Section S18 continued Warning feature responses

Table S18b. Separate Yes, No, and Not sure responses in the Saudi cohort.

Item	Feature	Study key	Yes, n (%)	No, n (%)	Not sure, n (%)
RF1	Age > 50 years with new onset of back pain	No	47 (35.9)	74 (56.5)	10 (7.6)
RF2	Age < 20 years with new onset of back pain	No	34 (26.0)	82 (62.6)	15 (11.5)
RF3	Personal history of cancer / malignancy	Yes	116 (88.5)	11 (8.4)	4 (3.1)
RF4	Unexplained weight loss (> 10% body weight in 3–6 months)	Yes	115 (87.8)	13 (9.9)	3 (2.3)
RF5	Constant, progressive, non-mechanical pain	Yes	87 (66.4)	22 (16.8)	22 (16.8)
RF6	Severe night pain unrelieved by position change or rest	Yes	102 (77.9)	19 (14.5)	10 (7.6)
RF7	History of fever or recent bacterial infection (e.g., UTI, skin)	Yes	99 (75.6)	22 (16.8)	10 (7.6)
RF8	Intravenous drug use	Yes	71 (54.2)	37 (28.2)	23 (17.6)
RF9	Immunosuppression (HIV, transplant, chronic corticosteroid use)	Yes	88 (67.2)	22 (16.8)	21 (16.0)
RF10	Significant trauma (proportional to age and bone health)	Yes	92 (70.2)	23 (17.6)	16 (12.2)
RF11	Saddle anesthesia / perineal numbness	Yes	104 (79.4)	15 (11.5)	12 (9.2)
RF12	Recent onset of bladder dysfunction (retention or overflow incontinence)	Yes	109 (83.2)	13 (9.9)	9 (6.9)
RF13	Recent onset of bowel incontinence	Yes	109 (83.2)	13 (9.9)	9 (6.9)
RF14	Bilateral lower-limb weakness or sensory loss	Yes	106 (80.9)	20 (15.3)	5 (3.8)
RF15	Progressive neurological deficit	Yes	99 (75.6)	19 (14.5)	13 (9.9)
RF16	Severe morning stiffness lasting > 1 hour (younger patient)	Yes	71 (54.2)	47 (35.9)	13 (9.9)
RF17	Pulsatile abdominal mass	Yes	103 (78.6)	16 (12.2)	12 (9.2)
RF18	Severe non-mechanical throbbing back pain in elderly with cardiovascular risk	Yes	103 (78.6)	13 (9.9)	15 (11.5)
RF19	Prolonged corticosteroid use	Yes	73 (55.7)	27 (20.6)	31 (23.7)
RF20	Failure of pain to respond to any treatment over > 4 weeks	No	87 (66.4)	23 (17.6)	21 (16.0)

RF1, RF2, and RF20 retain their submitted study keys for transparent exploration, but the compound investigation-or-referral stem prevents treating agreement as a validated test of standalone referral necessity. No and Not sure are shown separately even though both receive zero on positively keyed core items. No responses were excluded or imputed for this table.

Supplementary Section S19 Post hoc scoring sensitivity analyses

The targeted guideline-based appraisal in S10 motivated the scoring scenarios below. This revision-stage work was undertaken after collection, with submitted keys and descriptive results available; it is not prospective validation or an independent clinician assessment. The alternatives were examined as related exploratory checks, not selected as replacement keys according to favourable statistical results. Original responses, cohort allocation, predictors, and the V9–V12 primary outcome were unchanged. Only binary strict-vignette and specified awareness keys varied; graded and Jette-compatible scores were held fixed.

Table S19a. Alternative scoring rules and scope.

Scenario	Rule	Clinical interpretation
Baseline	V7: 2; V8: 2 or 3. RF1/RF2/RF20: No; RF3–RF19: Yes.	Submitted investigator-defined keys, not independently validated.
V7 alternative	Credit 2 or 3 for V7.	Medical review is supported; a uniquely superior concurrent-treatment sequence is not established.
V8 alternative	Credit 3 only for V8.	Stricter exploratory treatment-timing scenario; not a guideline-mandated correction.
Combined vignette alternatives	Apply both V7 and V8 alternatives.	Tests their joint influence on strict-key scores and secondary associations.
RF20 Yes	Credit Yes instead of No for RF20; Not sure remains 0.	Tests reassessment under the compound investigation-or-referral stem; not universal referral for all persistent pain.
Core 17	Exclude RF1, RF2 and RF20; retain RF3–RF19.	Existing positively keyed core score (0–17); changed range and content, not an alternative 20-item scale.

Analyses used the same 131 Saudi participants; nationality comparisons additionally used 12 non-Saudi participants. Awareness comparisons used Welch t tests, nominal 95% intervals, and a five-comparison Holm family within each score scenario. Hedges g permits descriptive comparison across score ranges but does not establish identical constructs. Nationality analyses retained the original 18-outcome Holm family within each scenario; Core17 was already one of those outcomes and was not duplicated. Confidence associations used exploratory Spearman tests with unadjusted p values; nominal percentile 95% intervals for rho and paired changes used 5,000 participant resamples (seed 20260926). No additional multiplicity adjustment across scoring scenarios was applied. No new regression of awareness or total vignette scores was fitted.

The main logistic outcome and six covariates were identical across all scenarios. Verification refits reproduced every coefficient and standard error exactly (maximum absolute change 0). Scope-model inputs were also unchanged. This is expected invariance because the altered items do not enter these models; it is not a sensitivity test of the primary V9–V12 key and is not an interaction test.

Supplementary Section S19 continued Awareness subgroup associations

Table S19b. Awareness subgroup contrasts under baseline and alternative scoring.

Score	Comparison	n group 1 / 0	Mean difference (95% CI)	Hedges g	Holm p
Baseline 20	Experience >=10 years	50 / 81	−0.32 (−1.73 to 1.08)	−0.08	0.650
Baseline 20	Formal screening training	106 / 25	1.70 (−0.52 to 3.93)	0.44	0.510
Baseline 20	Entry-level DPT	8 / 123	2.86 (1.64 to 4.09)	0.74	<0.001
Baseline 20	MSK/sports practice	64 / 67	0.70 (−0.64 to 2.04)	0.18	0.620
Baseline 20	Prior direct-access exposure	88 / 43	0.91 (−0.51 to 2.34)	0.23	0.620
RF20 Yes 20	Experience >=10 years	50 / 81	−0.37 (−1.87 to 1.14)	−0.09	0.813
RF20 Yes 20	Formal screening training	106 / 25	1.57 (−0.76 to 3.89)	0.38	0.714
RF20 Yes 20	Entry-level DPT	8 / 123	3.01 (1.27 to 4.75)	0.73	0.014
RF20 Yes 20	MSK/sports practice	64 / 67	0.60 (−0.83 to 2.03)	0.14	0.813
RF20 Yes 20	Prior direct-access exposure	88 / 43	1.02 (−0.50 to 2.53)	0.24	0.714
Core 17	Experience >=10 years	50 / 81	−0.51 (−1.92 to 0.91)	−0.13	0.738
Core 17	Formal screening training	106 / 25	1.65 (−0.48 to 3.77)	0.42	0.498
Core 17	Entry-level DPT	8 / 123	2.72 (1.18 to 4.26)	0.69	0.011
Core 17	MSK/sports practice	64 / 67	0.62 (−0.74 to 1.99)	0.16	0.738
Core 17	Prior direct-access exposure	88 / 43	0.92 (−0.52 to 2.37)	0.23	0.626

Group 1 has the named characteristic; group 0 does not. DPT compares 8 entry-level DPT with 123 Bachelor-entry respondents. Each scenario has a five-test Holm family. The core score has 17 rather than 20 items; do not compare its raw point contrast as if the scales were identical. All four non-DPT contrast intervals include zero in all scenarios. These subgroup results remain exploratory.

Supplementary Section S19 continued Change in awareness contrasts

Table S19c. Direct change in each group contrast after RF20 was keyed Yes.

The same participants contributed to both keys. Welch tests were applied to each participant's change in score to estimate the change in the corresponding between-group contrast. Nominal intervals all include zero; this does not establish equivalence. The original effect estimates, rather than p-value changes alone, should guide interpretation.

Comparison	Change in mean contrast	95% CI
Experience >=10 years	−0.05	−0.33 to 0.24
Formal screening training	−0.14	−0.44 to 0.17
Entry-level DPT	0.15	−0.48 to 0.77
MSK/sports practice	−0.10	−0.37 to 0.17
Prior direct-access exposure	0.10	−0.19 to 0.40

Supplementary Section S19 continued Confidence associations

Table S19d. Confidence correlations and paired changes from baseline.

Score family	Scenario	rho (95% CI)	Change in rho (95% CI)	Unadjusted p
Strict vignette	Baseline	0.275 (0.094 to 0.444)	Reference	0.001
Strict vignette	V7 alternative	0.270 (0.085 to 0.440)	−0.005 (−0.037 to 0.026)	0.002
Strict vignette	V8 alternative	0.268 (0.083 to 0.440)	−0.007 (−0.033 to 0.018)	0.002
Strict vignette	V7 and V8 alternatives	0.259 (0.071 to 0.429)	−0.017 (−0.062 to 0.030)	0.003
Awareness	Baseline	0.258 (0.078 to 0.420)	Reference	0.003
Awareness	RF20 Yes	0.279 (0.099 to 0.439)	0.020 (−0.011 to 0.054)	0.001
Awareness	Core 17	0.240 (0.058 to 0.407)	−0.018 (−0.064 to 0.026)	0.006

All n = 131. Intervals are participant-bootstrap percentile intervals; within each family, the same resample was used for baseline and alternative scores when calculating their difference. The paired-change intervals include zero for every tested alternative. Correlations remain weak, with no claim of equivalence, causal effects, or validation.

No nationality comparison met the 18-test within-scenario Holm threshold under the baseline, V7-only, V8-only, combined-vignette, RF20-Yes, or combined-vignette-plus-RF20 scenarios. The 12 non-Saudi participants limit precision; non-significance does not establish similarity. Complete 18-outcome results for all six scenarios are supplied in S9/post_hoc_scoring_sensitivity/results/07_nationality_comparisons.csv.

Reproduction and provenance

S11 is the separate portable R script. It uses S1 and the supplied S9 analysis dataset and reference tables, verifies the baseline scores/results, and writes every scenario result. The analysis was executed in R 4.6.1; execution dates, code commits, and input checksums are provided in S9. Source code, all result CSVs, session information, checks, and the portable-script verification record are included in S9/post_hoc_scoring_sensitivity/. The analysis does not overwrite the original S2 results.

Supplementary Section S20 Additional administration and descriptive details

Recruitment benchmark and administration

The pragmatic sample benchmark used $n = Z^2p(1 - p)/d^2$, with $Z = 1.96$, $p = 0.50$ and $d = 0.05$, giving 384.16, rounded to 385 (main reference 18). It was a recruitment target, not evidence of representativeness or population precision under convenience sampling. Invitation, unique-viewer and dissemination-site denominators were not recorded; reasons for non-participation are unknown.

The survey collected no names, emails, cookies, IP addresses or unique participant tokens. Google sign-in was not required, and no technical one-response restriction was applied. Unique visitors and duplicate participation could not be established. No incentives were offered. Items followed the fixed order in S5, with no randomisation or adaptive questioning beyond the two consent/eligibility gates. The survey had no separate review step before final submission; its browser-screen count was not recorded. Required scored and demographic/professional items, including certification, were complete among the 143 eligible respondents. The open-text question was optional. No completion-time exclusion was applied, and no weighting or propensity adjustment was used.

Processing and open-text review

Text encoding and response labels were standardised with checks against silent miscoding. Exact timestamps were removed from the shared dataset; study identifiers and calendar dates were retained. The certification processing correction and four contradictory co-selections are documented in Section S17 and S9; there was no imputation or participant exclusion. The author's manual checks compared age with post-licensure experience and highest qualification and identified no inconsistency requiring exclusion. Formal identical-pattern and low-engagement checks were not performed.

Eight open-text themes were defined before reading comments. During revision, all 32 substantive comments were checked by the author against assigned labels. Improved word boundaries, limited spelling normalisation and English translation of Arabic text supported review; keyword matches were candidate flags. Final decisions and rationales were explicitly encoded in R. The author interpreted "It's mandatory in next decade" as referring to future mandatory direct access, and "Pathway for certain diagnosis like knee OA, tennis elbow, trigger finger..ETC" as referral pathways. These interpretations and remaining ambiguities are distinguished from the original wording in S8. Multiple labels were permitted. There was no independent second coder or independent translation check. Frequencies describe mentions within the 32 substantive comments, not relative importance or population prevalence.

Additional statistical detail

Model diagnostics covered age functional form, multicollinearity, influence, apparent discrimination, prediction error, descriptive calibration and heteroscedasticity (Tables S10–S12). Main logistic intervals are nominal 95% Wald intervals; the original Firth analysis uses penalised profile-likelihood intervals, while expanded mean-bias-reduced models use Wald intervals. Full post hoc scoring methods, including 5,000 paired participant-bootstrap resamples and seed 20260926, are retained in Section S19.

Supplementary Section S20 continued Additional numerical summaries

Table S20a. Additional descriptive and internal-consistency summaries for the Saudi cohort (n = 131).

Measure	Result	Interpretation or source
Number of urgent/critical vignettes referred first	0: 18; 1: 5; 2: 5; 3: 26; 4: 77 participants	Figure 2; File S9, output 37. The remaining 54 include concurrent-referral responses.
Treatment without referral for all four urgent/critical vignettes	9/131; 6.9% (95% Wilson CI 3.7–12.5)	Hypothetical responses, not observed clinical actions.
Non-referral-first vignette count (0–4)	Mean 0.94; SD 1.42	Complement of referral-first count; not a clinical error count.
Strict-key agreement (0–12)	Mean 7.83; SD 2.48; median 8; IQR 6–10	65.3% of available points; submitted investigator-defined key.
Study-defined graded score (0–24)	Mean 18.02; SD 4.25; median 19; IQR 16–21	Category percentages: musculoskeletal 73.8%; medically concerning 76.0%; urgent/critical 76.5%.
Jette-compatible score (0–12)	Mean 10.18; SD 2.06	Category percentages appear in Table S8. Different vignette sets prevent validation by comparison.
Confidence with total awareness	Spearman ρ = 0.26; unadjusted p = 0.003	Exploratory weak association; File S9, output 15 (Spearman correlations).
Confidence with strict-key agreement	Spearman ρ = 0.28; unadjusted p = 0.001	Exploratory weak association; scenario-specific intervals in S19.
Confidence with referral-first vignette count	Spearman ρ = 0.24; unadjusted p = 0.005	Exploratory weak association.
Total awareness with screening-frequency score	Spearman ρ = 0.28; unadjusted p = 0.001	Recognition and recalled screening are different constructs.
Internal consistency: total/core awareness	KR-20 = 0.793 / 0.852	Descriptive consistency within the 20-item and 17-item sets, respectively.
Internal consistency: stakeholder support, barriers, preparation need	Raw Cronbach's α = 0.904, 0.901, 0.881	Does not establish broader questionnaire reliability or validity.
Vignette item-rest correlations	V7: −0.064; V8: 0.120	Vignette alpha was not calculated because vignettes represent heterogeneous presentations; correlations do not validate clinical keys.

Values are descriptive or exploratory; p values in this table are unadjusted. Complete source results are in S9. Internal consistency and item-rest statistics are not evidence of independent clinical validation.

Supplementary Figure S1 Adjusted referral model

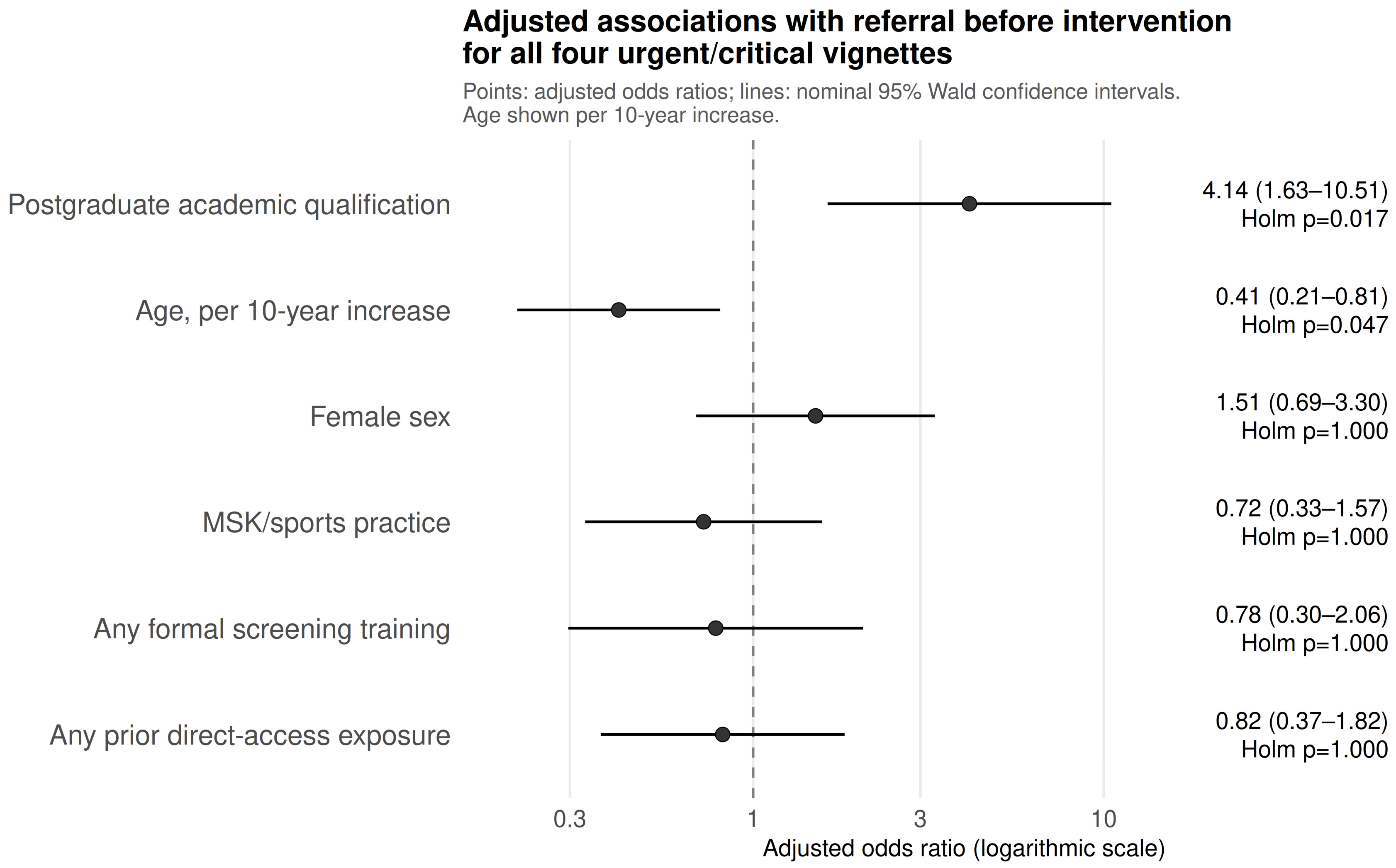

Supplementary Figure S1. Adjusted associations with referral before intervention for all four urgent/critical vignettes (n = 131). Points represent odds ratios and lines nominal 95% Wald confidence intervals on a logarithmic scale. Age is shown per 10-year increase; Table 2 reports the same model per year. Reference categories are male sex, no postgraduate academic qualification, other clinical areas, no formal screening training, and no prior direct-access exposure. All symbols use one colour; Holm-adjusted p values are printed for all six coefficients. These are exploratory associations, not a validated prediction model. The plot was generated in R using S2. MSK, musculoskeletal.
