# Supplementary material for: Clinical Decision Making, Red-Flag Recognition, and Scope-of-Practice Perspectives for Direct Access Physical Therapy in Saudi Arabia
Source: Healthcare (Basel). 2026 Oct 8;14(19):3347. doi: 10.3390/healthcare14193347 (PMC13664920; doi:10.3390/healthcare14193347)
Supplement: Supplementary file 1 [file healthcare-14-03347-s001.zip › File_S5_Complete_Questionnaire.pdf]

Supplementary File S5

Clinical Decision-Making, Red-Flag Recognition, and Scope-of-Practice Perspectives for Direct Access Physical Therapy in Saudi Arabia

Saudi Physical Therapists' Readiness for Direct Access - National Survey

Participant Information Sheet

Study title: Clinical Decision-Making, Red-Flag Recognition, and Scope-of-Practice Perspectives for Direct Access Physical Therapy in Saudi Arabia.

Principal Investigator: Msaad Alzhrani, PT, PhD - Department of Physical Therapy and Rehabilitation, College of Applied Medical Sciences, Majmaah University.

Purpose: This study assesses clinical decision-making, red-flag knowledge and screening, regulatory understanding, scope perspectives, and implementation conditions relevant to direct-access practice among physical therapists in Saudi Arabia using an investigator-developed, literature-informed multidomain questionnaire that was not formally validated as a complete instrument. Findings will inform regulatory, educational, and policy decisions aligned with Vision 2030.

Who may participate: Licensed physical therapists registered with the Saudi Commission for Health Specialties (SCFHS), currently practicing in Saudi Arabia, with at least one year of post-licensure clinical experience.

Time required: Approximately 15-20 minutes. You may save your progress and return later using the same device/browser.

Confidentiality: The survey is fully anonymous. No identifying information will be collected, stored, or published. Aggregated data will be reported only at the group level.

Voluntary participation: Your participation is entirely voluntary. You may withdraw at any time before submission with no consequences.

Ethics: This study has been approved by the Institutional Review Board (IRB) of Majmaah University and conforms to the Declaration of Helsinki and the Saudi National Committee of Bioethics (NCBE) guidelines.

Contact: For questions, please contact the PI at Majmaah University, College of Applied Medical Sciences.

* Indicates a required question.

Informed Consent

Before continuing, please read the Participant Information Sheet above and indicate your consent below. Selecting 'No' will end the survey.

Q1. INFORMED CONSENT - I confirm that I have read and understood the Participant Information Sheet, that I am at least 18 years old, that my participation is voluntary, and that I consent to participate in this study. *

- Yes - I consent to participate.
- No - I do not consent to participate.

Professional Eligibility

Q2. INCLUSION CRITERIA - I confirm that I meet ALL of the following:
• I am a licensed physical therapist registered with the Saudi Commission for Health Specialties (SCFHS).
• I am currently practicing physical therapy clinically in the Kingdom of Saudi Arabia (any setting).
• I have at least one (1) year of post-licensure clinical experience.
• I am NOT an intern, student, or trainee.
• I am NOT working exclusively in non-clinical academic or administrative roles.

- Yes - I meet ALL of the above inclusion criteria.
- No - I do NOT meet all of the above criteria.

Selecting 'No' ended the survey.

Section S1 - Demographic and Professional Characteristics

These questions capture background information used for descriptive and stratified analyses only. No identifying data are collected.

1.1 Age (in years) *

1.2 Sex *

- Male
- Female

1.3 Nationality *

- Saudi
- Non-Saudi

1.4 Administrative region where you currently practice *

- Riyadh Region
- Makkah Region
- Madinah Region
- Eastern Province
- Qassim Region
- Asir Region
- Tabuk Region
- Hail Region
- Northern Borders Region
- Jazan Region
- Najran Region
- Al-Bahah Region
- Al-Jouf Region

1.5 Primary practice sector *

- Ministry of Health (MoH) hospital/clinic
- Ministry of Defence / Military Medical Services
- Ministry of Interior medical services
- National Guard Health Affairs (NGHA)
- Academic medical center / University hospital
- Private hospital
- Private clinic / polyclinic
- Specialized rehabilitation center
- Sports / fitness facility
- Home-based / community care
- Other

1.6 Highest academic qualification in physical therapy *

- Bachelor's degree (BSc / BPT)
- Doctor of Physical Therapy (DPT)
- Master's degree (MSc / MPT)
- PhD / DSc
- Other

1.7 Entry-level physical therapy degree *

- Bachelor's degree of Physical Therapy (PT)
- Doctor of Physical Therapy (DPT)

1.8 Years of post-licensure clinical experience *

- 1 to <3 years
- 3 to <6 years
- 6 to <10 years
- 10 to <15 years
- 15 to <20 years
- 20 years or more

1.9 Primary clinical area of practice *

- Musculoskeletal / Orthopedic

- Neurological
- Cardiopulmonary
- Pediatric
- Sports physical therapy
- Geriatric
- Women's health
- Acute care / ICU
- General / mixed practice
- Other

1.10 Do you hold any of the following postgraduate certifications? (select all that apply) *

- Saudi Board of Physical Therapy
- Orthopedic Manual Therapy / OMPT certification
- Sports specialty certification
- Neurological specialty certification
- Pediatric specialty certification
- Other clinical specialty certification
- None

1.11 Have you received formal training in medical screening / differential diagnosis (e.g., a course, module, or workshop) at any point during your career? (select all that apply) *

- Yes - during entry-level (undergraduate) education
- Yes - during postgraduate / specialty training
- Yes - through an external CPD course / workshop
- Yes - through self-directed study
- No formal training

1.12 Have you ever practiced in a setting where direct access (self-referral) was permitted? *

- Yes - currently practicing under direct access
- Yes - previously, outside Saudi Arabia
- Yes - limited / partial direct access in Saudi Arabia
- No - never

Section S2 - Clinical Vignettes (Decision-Making)

For each of the following twelve clinical scenarios, indicate the management decision that you believe is most appropriate based ONLY on the information provided. Please select ONE option per vignette.

Vignette V1: A 35-year-old female accountant presents with a 3-week history of right lateral elbow pain that began after extensive gardening. Pain is reproduced by resisted wrist extension and direct palpation over the lateral epicondyle. There is no radiation, no neurological deficit, and no constitutional symptoms (no fever, weight loss, fatigue). Past medical history is unremarkable.
What is the most appropriate management decision? *

- 1) TREAT - Proceed with physical therapy intervention WITHOUT medical referral.
- 2) TREAT AND CONSULT/REFER - Provide physical therapy intervention AND concurrently consult with or refer to a medical professional.
- 3) REFER FIRST - Refer the patient to a medical professional BEFORE any physical therapy intervention.

Vignette V2: A 28-year-old male recreational runner reports a 2-week history of anterior right knee pain. Pain worsens with descending stairs and after prolonged sitting (positive 'theatre sign'). There is no effusion, no instability, and no locking. McConnell's test is positive. Vital signs and general health review are normal.
What is the most appropriate management decision? *

- 1) TREAT - Proceed with physical therapy intervention WITHOUT medical referral.
- 2) TREAT AND CONSULT/REFER - Provide physical therapy intervention AND concurrently consult with or refer to a medical professional.
- 3) REFER FIRST - Refer the patient to a medical professional BEFORE any physical therapy intervention.

Vignette V3: A 45-year-old male warehouse worker presents with mechanical low back pain that began 5 days ago after lifting a heavy box. Pain is localized to the lumbar paraspinal region with no radicular symptoms, no red flags, and no constitutional symptoms. Pain is worse with flexion and eased by lying supine.
What is the most appropriate management decision? *

- 1) TREAT - Proceed with physical therapy intervention WITHOUT medical referral.
- 2) TREAT AND CONSULT/REFER - Provide physical therapy intervention AND concurrently consult with or refer to a medical professional.
- 3) REFER FIRST - Refer the patient to a medical professional BEFORE any physical therapy intervention.

Vignette V4: A 52-year-old non-diabetic female presents with progressive right shoulder stiffness over 4 months. She reports night pain only when lying on the affected side. Active and passive range of motion are equally restricted in all planes (capsular pattern). No history of trauma. No constitutional symptoms or weight loss.
What is the most appropriate management decision? *

- 1) TREAT - Proceed with physical therapy intervention WITHOUT medical referral.
- 2) TREAT AND CONSULT/REFER - Provide physical therapy intervention AND concurrently consult with or refer to a medical professional.
- 3) REFER FIRST - Refer the patient to a medical professional BEFORE any physical therapy intervention.

Vignette V5: A 30-year-old healthy female complains of bilateral plantar heel pain, worse with the first steps in the morning and after prolonged standing. She is otherwise healthy. There is local tenderness over the medial calcaneal tubercle, no swelling, no neurological findings, and no systemic symptoms.
What is the most appropriate management decision? *

- 1) TREAT - Proceed with physical therapy intervention WITHOUT medical referral.
- 2) TREAT AND CONSULT/REFER - Provide physical therapy intervention AND concurrently consult with or refer to a medical professional.
- 3) REFER FIRST - Refer the patient to a medical professional BEFORE any physical therapy intervention.

Vignette V6: A 40-year-old male desk worker reports right-sided neck pain radiating to the lateral upper arm for 10 days. Spurling's test is positive. Strength, deep tendon reflexes, and sensation are intact throughout the upper limb. No bowel/bladder symptoms, no fever, no weight loss.
What is the most appropriate management decision? *

- 1) TREAT - Proceed with physical therapy intervention WITHOUT medical referral.
- 2) TREAT AND CONSULT/REFER - Provide physical therapy intervention AND concurrently consult with or refer to a medical professional.
- 3) REFER FIRST - Refer the patient to a medical professional BEFORE any physical therapy intervention.

Vignette V7: A 60-year-old male with type 2 diabetes for 15 years presents with bilateral foot numbness and tingling that has been progressing over the past 6 months. He reports occasional unsteadiness when walking. He has not seen his physician for 2 years. There is no fever, no skin lesions, and peripheral pulses are present.
What is the most appropriate management decision? *

- 1) TREAT - Proceed with physical therapy intervention WITHOUT medical referral.
- 2) TREAT AND CONSULT/REFER - Provide physical therapy intervention AND concurrently consult with or refer to a medical professional.
- 3) REFER FIRST - Refer the patient to a medical professional BEFORE any physical therapy intervention.

Vignette V8: A 55-year-old female presents with right shoulder pain for 3 weeks. She also reports increased fatigue and intermittent palpitations that she attributes to stress. She has no chest pain. Shoulder examination is consistent with subacromial impingement, but her cardiac history is unclear and she has not seen a physician recently.
What is the most appropriate management decision? *

- 1) TREAT - Proceed with physical therapy intervention WITHOUT medical referral.
- 2) TREAT AND CONSULT/REFER - Provide physical therapy intervention AND concurrently consult with or refer to a medical professional.
- 3) REFER FIRST - Refer the patient to a medical professional BEFORE any physical therapy intervention.

Vignette V9: A 68-year-old female with osteoarthritis presents with progressive bilateral knee pain. She mentions that she has been taking ibuprofen daily for 6 months and now reports occasional dark stools and increasing fatigue. She has no abdominal pain. Vitals show mild tachycardia.
What is the most appropriate management decision? *

- 1) TREAT - Proceed with physical therapy intervention WITHOUT medical referral.
- 2) TREAT AND CONSULT/REFER - Provide physical therapy intervention AND concurrently consult with or refer to a medical professional.
- 3) REFER FIRST - Refer the patient to a medical professional BEFORE any physical therapy intervention.

Vignette V10: A 38-year-old male presents with severe lower back pain for 3 days. He reports new-onset urinary retention, perineal numbness ('saddle anesthesia'), and bilateral lower limb weakness. There is reduced perianal sensation on examination.
What is the most appropriate management decision? *

- 1) TREAT - Proceed with physical therapy intervention WITHOUT medical referral.
- 2) TREAT AND CONSULT/REFER - Provide physical therapy intervention AND concurrently consult with or refer to a medical professional.
- 3) REFER FIRST - Refer the patient to a medical professional BEFORE any physical therapy intervention.

Vignette V11: A 72-year-old male with a history of hypertension and smoking presents with sudden-onset, severe, throbbing lower back pain radiating to the abdomen. The pain is NOT related to position or movement. On examination, a pulsatile abdominal mass is palpated and blood pressure is 165/95 mmHg.
What is the most appropriate management decision? *

- 1) TREAT - Proceed with physical therapy intervention WITHOUT medical referral.
- 2) TREAT AND CONSULT/REFER - Provide physical therapy intervention AND concurrently consult with or refer to a medical professional.
- 3) REFER FIRST - Refer the patient to a medical professional BEFORE any physical therapy intervention.

Vignette V12: A 45-year-old male with a history of intravenous drug use presents with 2 weeks of severe lower back pain, fever (38.6 °C), night sweats, and pain that is worse at night and unrelieved by rest. He has lost 4 kg in the past month.
What is the most appropriate management decision? *

- 1) TREAT - Proceed with physical therapy intervention WITHOUT medical referral.
- 2) TREAT AND CONSULT/REFER - Provide physical therapy intervention AND concurrently consult with or refer to a medical professional.
- 3) REFER FIRST - Refer the patient to a medical professional BEFORE any physical therapy intervention.

Section S3 - Red Flags Knowledge

Red flags are clinical features that may indicate serious underlying pathology (e.g., malignancy, fracture, infection, cauda equina syndrome, abdominal aortic aneurysm). This section assesses your AWARENESS of each red flag and how frequently you SCREEN for it during initial assessment.

3.1 AWARENESS - For each item below, indicate whether you consider it a RED FLAG warranting further investigation or referral. *

Item	Yes - it is a red flag	No - not a red flag	Not sure
Age > 50 years with new onset of back pain	○	○	○
Age < 20 years with new onset of back pain	○	○	○
Personal history of cancer / malignancy	○	○	○
Unexplained weight loss (> 10% body weight in 3-6 months)	○	○	○
Constant, progressive, non-mechanical pain	○	○	○
Severe night pain unrelieved by position change or rest	○	○	○
History of fever or recent bacterial infection (e.g., UTI, skin)	○	○	○
Intravenous drug use	○	○	○
Immunosuppression (HIV, transplant, chronic corticosteroid use)	○	○	○
Significant trauma (proportional to age and bone health)	○	○	○
Saddle anesthesia / perineal numbness	○	○	○
Recent onset of bladder dysfunction (retention or overflow incontinence)	○	○	○
Recent onset of bowel incontinence	○	○	○
Bilateral lower-limb weakness or sensory loss	○	○	○
Progressive neurological deficit	○	○	○
Severe morning stiffness lasting > 1 hour (younger patient)	○	○	○
Pulsatile abdominal mass	○	○	○
Severe non-mechanical throbbing back pain in elderly with cardiovascular risk	○	○	○
Prolonged corticosteroid use	○	○	○
Failure of pain to respond to any treatment over > 4 weeks	○	○	○

3.2 UTILIZATION - How frequently do you SCREEN for each of the following during your initial patient assessment? *

Item	Never	Rarely	Sometimes	Often	Always
Age > 50 years with new onset of back pain	○	○	○	○	○
Age < 20 years with new onset of back pain	○	○	○	○	○
Personal history of cancer / malignancy	○	○	○	○	○
Unexplained weight loss (> 10% body weight in 3-6 months)	○	○	○	○	○
Constant, progressive, non-mechanical pain	○	○	○	○	○
Severe night pain unrelieved by position change or rest	○	○	○	○	○
History of fever or recent bacterial infection (e.g., UTI, skin)	○	○	○	○	○
Intravenous drug use	○	○	○	○	○
Immunosuppression (HIV, transplant, chronic corticosteroid use)	○	○	○	○	○
Significant trauma (proportional to age and bone health)	○	○	○	○	○
Saddle anesthesia / perineal numbness	○	○	○	○	○
Recent onset of bladder dysfunction (retention or overflow incontinence)	○	○	○	○	○
Recent onset of bowel incontinence	○	○	○	○	○
Bilateral lower-limb weakness or sensory loss	○	○	○	○	○
Progressive neurological deficit	○	○	○	○	○
Severe morning stiffness lasting > 1 hour (younger patient)	○	○	○	○	○
Pulsatile abdominal mass	○	○	○	○	○
Severe non-mechanical throbbing back pain in elderly with cardiovascular risk	○	○	○	○	○
Prolonged corticosteroid use	○	○	○	○	○
Failure of pain to respond to any treatment over > 4 weeks	○	○	○	○	○

3.3 Overall, how confident are you in identifying red flags during initial assessment? *

- 1 - Not at all confident
- 2
- 3
- 4
- 5 - Extremely confident

Section S4 - Scope of Practice and Direct Access

This section explores your understanding of, and views on, the scope of physical therapy practice for direct access in Saudi Arabia.

4A. Awareness of legal status

4.1 Are you aware of the current regulations governing direct access to physical therapy in Saudi Arabia? *

- Yes, fully aware
- Yes, partially aware
- No, not aware

4.2 In your understanding, is direct access to physical therapy currently permitted in Saudi Arabia? *

- Yes - fully permitted
- Yes - partially permitted (limited settings)
- No - not permitted
- I am not sure

4B. Endorsed scope of practice for direct access

4.3 In your view, the legal scope of physical therapy practice in Saudi Arabia SHOULD include each of the following: *

Item	Strongly Disagree	Disagree	Neutral	Agree	Strongly Agree
**Initial assessment and clinical diagnosis without prior physician referral**	○	○	○	○	○
**Ordering plain radiographs (X-ray)**	○	○	○	○	○
**Ordering advanced imaging (MRI, CT)**	○	○	○	○	○
**Ordering laboratory investigations**	○	○	○	○	○
**Direct referral to medical specialists**	○	○	○	○	○
**Prescribing medications for musculoskeletal conditions**	○	○	○	○	○
**Performing therapeutic injections (e.g., trigger-point, joint)**	○	○	○	○	○
**Discharging patients without physician sign-off**	○	○	○	○	○
**Issuing sick-leave / fitness-for-work certificates**	○	○	○	○	○

4C. Perceived support from stakeholders

4.4 How supportive do you perceive each of the following stakeholders to be regarding direct access to physical therapy in Saudi Arabia? *

Item	Strongly Opposed	Opposed	Neutral	Supportive	Strongly Supportive
**Saudi Physical Therapy Association**	○	○	○	○	○
**Saudi Commission for Health Specialties (SCFHS)**	○	○	○	○	○
**Ministry of Health policymakers**	○	○	○	○	○
**Physicians**	○	○	○	○	○
**The Saudi public**	○	○	○	○	○
**Medical insurance providers**	○	○	○	○	○
**Hospital / clinic administrators**	○	○	○	○	○

4D. Educational adequacy

4.5 Indicate your level of agreement with each statement: *

Item	Strongly Disagree	Disagree	Neutral	Agree	Strongly Agree
Entry-level physical therapy education in Saudi Arabia adequately prepares graduates for direct access.	○	○	○	○	○
Postgraduate specialty training should be required for direct access practice.	○	○	○	○	○
Continuous professional development (CPD) requirements should be increased to support direct access.	○	○	○	○	○
A formal credentialing pathway is needed for first-contact / direct-access PTs in Saudi Arabia.	○	○	○	○	○

4E. Perceived barriers

4.6 In your view, how IMPORTANT is each of the following as a barrier to implementing direct access to physical therapy in Saudi Arabia? *

Item	Not a barrier	Minor barrier	Moderate barrier	Major barrier	Critical barrier
Lack of supportive legal / regulatory framework	○	○	○	○	○
Limited support from physicians	○	○	○	○	○
Limited support from policymakers	○	○	○	○	○
Limited public awareness of physical therapy	○	○	○	○	○
Insurance / reimbursement issues	○	○	○	○	○
Inadequate entry-level education for direct access	○	○	○	○	○
Lack of postgraduate / specialty training pathways	○	○	○	○	○
Concerns about patient safety	○	○	○	○	○
Medico-legal liability concerns	○	○	○	○	○
Limited evidence-based practice culture	○	○	○	○	○
Cultural / institutional resistance	○	○	○	○	○

4.7 (Optional) Please describe any specific training, support, or policy changes you feel are most important to enable direct access practice in Saudi Arabia.

____

____

____

____
