# Supplementary material for: Clinical Decision Making, Red-Flag Recognition, and Scope-of-Practice Perspectives for Direct Access Physical Therapy in Saudi Arabia
Source: Healthcare (Basel). 2026 Oct 8;14(19):3347. doi: 10.3390/healthcare14193347 (PMC13664920; doi:10.3390/healthcare14193347)
Supplement: Supplementary file 1 [file healthcare-14-03347-s001.zip › File_S6_STROBE_Checklist.pdf]

Supplementary File S6 - Completed STROBE Checklist

Clinical Decision-Making, Red-Flag Recognition, and Scope-of-Practice Perspectives for Direct Access Physical Therapy in Saudi Arabia

Section	Item	STROBE recommendation	Reported in manuscript
Title and abstract	1a	Indicate the study design with a commonly used term in the title or abstract.	Abstract (cross-sectional survey design stated).
Title and abstract	1b	Provide an informative and balanced abstract summary of what was done and found.	Abstract.
Introduction	2	Explain the scientific background and rationale.	Sections 1.1–1.3.
Introduction	3	State specific objectives, including any prespecified hypotheses.	Section 1.3; no directional hypotheses. Section 2.4 and S4 Table S16a distinguish the report's primary outcome from prospectively documented decisions.
Methods	4	Present key elements of the study design early in the paper.	Section 2.1.
Methods	5	Describe setting, locations, and relevant dates, including recruitment and data-collection periods.	Sections 2.1–2.2.
Methods	6a	Give eligibility criteria and sources/methods of participant selection.	Section 2.2.
Methods	7	Clearly define outcomes, exposures, predictors, potential confounders, and effect modifiers.	Sections 2.3–2.4; principal component items, response options, score direction and range are summarized in the main text. Supplementary File S4, Tables S1–S5 and S16b.
Methods	8	For each variable, give data sources and measurement/assessment methods.	Sections 2.3–2.5; Supplementary Files S4–S5.
Methods	9	Describe efforts to address potential sources of bias.	Sections 2.2 and 2.5 (sampling, eligibility and manual demographic review); Sections 2.4–2.6 (decision timing, exploratory analyses and sensitivities); Section 4.6 (measurement and selection limits).
Methods	10	Explain how the study size was arrived at.	Section 2.2 and S4 Section S20 retain the pragmatic recruitment benchmark of 385, its formula and assumptions, and the limitation of convenience sampling.
Methods	11	Explain how quantitative variables were handled, including groupings.	Sections 2.4–2.6; Table 1; Supplementary File S4, Tables S4–S5 and S16–S17. Age is numeric; experience is categorical in sensitivity models.
Methods	12a	Describe all statistical methods, including methods used to control for confounding.	Section 2.6; Table 2; Supplementary File S4, Sections S5, S10–S12, S17 and S19; S11 separate scoring-sensitivity code. Main and sensitivity estimator/interval definitions are distinguished.
Methods	12b	Describe methods used to examine subgroups and interactions.	Sections 2.4 and 2.6; exploratory subgroup/nationality comparisons and post hoc pooled, expanded-covariate and age–experience sensitivity analyses.
Methods	12c	Explain how missing data were addressed.	Sections 2.5–2.6: required scored items and demographic/professional responses were complete; no imputation. Literal None certification responses were restored from the original export after a processing error was identified; no participant was excluded (S4 Section S17).
Methods	12d	If applicable, describe analytical methods taking account of sampling strategy.	Section 2.2; S4 Section S20; Section 4.6: voluntary convenience sample, no weighting or propensity adjustment, and no claim of national representativeness.
Methods	12e	Describe any sensitivity analyses.	Sections 2.4 and 2.6; original Firth and V7 sensitivities plus post hoc age–experience, expanded-covariate, all-eligible descriptive, and alternative-scoring analyses; S4 Sections S11, S15, S17–S19; S10 appraisal and S11 code.
Results	13a	Report numbers of individuals at each stage of the study.	Section 3.1 and Figure 1.

Section	Item	STROBE recommendation	Reported in manuscript
Results	13b	Give reasons for non-participation at each stage.	Section 3.1 and Figure 1: non-consent and failure to confirm eligibility at mandatory gates. Invitation denominator and reasons for nonparticipation unavailable.
Results	13c	Consider use of a flow diagram.	Figure 1, generated in R from consent, eligibility and nationality responses using S2.
Results	14a	Give characteristics of study participants and information on exposures/potential confounders.	Table 1.
Results	14b	Indicate number of participants with missing data for each variable of interest.	Sections 2.2 and 2.5; S4 Section S17. Required scored and demographic/professional responses were complete. Certification responses were 55 None only, 72 named certification(s) only, and four named certification(s) plus None among 131 Saudi participants; zero were missing. No imputation.
Results	14c	Summarise follow-up time, if applicable.	Not applicable (cross-sectional survey).
Results	15	Report numbers of outcome events or summary measures.	Sections 3.2–3.7, Tables 2–3 and Figures 2–5; S4 Section S20 and Supplementary Figure S1.
Results	16a	Give unadjusted estimates and, if applicable, confounder-adjusted estimates with precision; clarify confounders included.	Section 3.3 and Table 2; explicit reference groups, effect estimates, nominal confidence intervals and Holm-adjusted p values; S4 Tables S11 and S17.
Results	16b	Report category boundaries when continuous variables were categorised.	Table 1; analytical coding detailed in Supplementary File S4.
Results	16c	If relevant, consider translating relative risk estimates into absolute risk for a meaningful period.	Not applicable to this cross-sectional analysis.
Results	17	Report other analyses, including subgroup and sensitivity analyses.	Sections 3.3, 3.5 and 3.7; S4 Sections S6, S10–S20; S8 open-text audit; S9 complete results; S10–S11 appraisal/code.
Discussion	18	Summarise key results with reference to study objectives.	Section 4.1.
Discussion	19	Discuss limitations, taking account of potential bias or imprecision.	Section 4.6; sample-size and precision limits, measurement/reference-standard uncertainty, decision chronology, self-report, language, model dependence, and no independent coding.
Discussion	20	Give a cautious overall interpretation considering objectives, limitations, multiplicity, and other evidence.	Sections 4.2–4.6.
Discussion	21	Discuss generalisability of the study results.	Sections 4.5–5.
Other information	22	Give the source of funding and role of funders.	Funding statement identifies the funding body and states its lack of involvement. The project number will be supplied upon acceptance during proofreading.

Checklist source: STROBE Statement - checklist for cross-sectional studies (www.strobe-statement.org).
