# Supplementary material for: Clinical Decision Making, Red-Flag Recognition, and Scope-of-Practice Perspectives for Direct Access Physical Therapy in Saudi Arabia
Source: Healthcare (Basel). 2026 Oct 8;14(19):3347. doi: 10.3390/healthcare14193347 (PMC13664920; doi:10.3390/healthcare14193347)
Supplement: Supplementary file 1 [file healthcare-14-03347-s001.zip › File_S7_CHERRIES_Checklist.pdf]

Supplementary File S7 - Completed CHERRIES Checklist

Clinical Decision-Making, Red-Flag Recognition, and Scope-of-Practice Perspectives for Direct Access Physical Therapy in Saudi Arabia

Category	Checklist item	CHERRIES reporting recommendation	Reported in manuscript
Design	Describe survey design	Target population, sampling frame and convenience/open-survey design.	Sections 2.1–2.2: national-scope multi-regional open internet survey; voluntary convenience sampling.
IRB and consent	IRB approval	State whether the study was approved by an IRB/ethics committee.	Section 2.7; Institutional Review Board Statement.
IRB and consent	Informed consent	Describe informed-consent process and participant information.	Sections 2.2 and 2.7; complete consent wording in Supplementary File S5.
IRB and consent	Data protection	Describe protection of personal information.	Sections 2.2 and 2.5; S4 Section S20: no direct identifiers, emails, cookies, IP addresses or unique participant tokens collected; timestamps removed from shared dataset.
Development and pre-testing	Development and testing	Describe survey development and any usability/technical testing before fielding.	Section 2.3: investigator-developed, literature-informed questionnaire; no independent prelaunch appraisal. Completed pilot, cognitive interviewing, or technical-usability records are not documented. Section 4.6 and S10: post hoc guideline-based appraisal during revision; no independent clinician appraisal or validation was obtained.
Recruitment	Open versus closed survey	State whether the survey was open or closed.	Sections 2.1–2.2: open Google Forms survey accessible to anyone with the link.
Recruitment	Contact mode	Indicate how potential participants were initially contacted.	Section 2.2: survey link distributed through professional associations, institutional contacts and professional networks.
Recruitment	Advertising the survey	Describe how/where the survey was announced.	Section 2.2: professional associations, institutional contacts and professional networks across intended clinical sectors/regions; exact dissemination-site count was not recorded.
Survey administration	Web/E-mail	State the type of e-survey and data capture method.	Section 2.1: English-language open Google Forms questionnaire.
Survey administration	Context	Describe the setting/context in which the survey link was distributed.	Sections 2.2–2.3: professional-network recruitment; the introductory message, not reproduced in S5, asked respondents to draw on workplace rules and experience. The vignette instructions separately required use of the supplied clinical information.
Survey administration	Mandatory/voluntary	State whether participation was mandatory or voluntary.	Sections 2.2 and 2.7: participation was voluntary; required scored items followed consent and eligibility gates.
Survey administration	Incentives	State whether incentives were offered.	S4 Section S20: no incentives were offered.
Survey administration	Time/Date	Report data-collection timeframe.	Sections 2.1 and 2.7: data collection from 11 May to 25 July 2026.
Survey administration	Randomization of items/questionnaires	State whether item/order randomization was used.	No item/order randomization was used; the questionnaire was administered in the fixed structure reproduced in Supplementary File S5.
Survey administration	Adaptive questioning	Describe conditional/adaptive questioning.	Section 2.2: Q1/Q2 were mandatory gates; selecting No terminated the form. No other adaptive questioning was used.
Survey administration	Number of items	Report number/structure of questionnaire items.	Section 2.3; detailed structure in Supplementary File S4, Table S7; complete questionnaire in S5.
Survey administration	Number of screens/pages	Report the number of browser screens/pages.	Not recorded as a browser-screen count; complete questionnaire content is provided in Supplementary File S5.

Category	Checklist item	CHERRIES reporting recommendation	Reported in manuscript
Survey administration	Completeness check	Describe completeness enforcement.	Sections 2.2 and 2.5: required scored items and demographic/professional responses, including the required certification item, were complete among eligible records. Optional comments were not required.
Survey administration	Review step	State whether respondents could review/change answers before submission.	S4 Section S20: the survey did not include a separate review step before final submission.
Response metrics	Unique site visitor	Explain how unique visitors were identified.	Section 2.2 and S4 Section S20: unique visitors could not be identified because cookies, IP addresses, and unique participant tokens were not collected.
Response metrics	View rate	Report/define view rate if available.	Sections 2.2 and 3.1: invitation/view denominator not recorded; conventional response/view rate unavailable.
Response metrics	Participation rate	Report/define participation rate if available.	Sections 2.2 and 3.1: invitation/consent-page viewer denominator not recorded; participation rate unavailable.
Response metrics	Completion rate	Report/define completion rate if available.	Sections 2.2 and 3.1: all 143 eligible respondents had complete required scored items; the viewer-based completion rate cannot be determined because viewers were not counted.
Multiple entries	Cookies used	State whether cookies were used to prevent/identify duplicate entries.	Section 2.2 and S4 Section S20: cookies were not collected or used.
Multiple entries	IP check	State whether IP addresses were used to prevent/identify duplicate entries.	Section 2.2 and S4 Section S20: IP addresses were not collected or used.
Multiple entries	Log file analysis	Describe other log-file duplicate checks.	Sections 2.2 and 4.6: no identifiers, tokens, or technical one-response restriction permitting conclusive duplicate determination. Similar response patterns would be flags, not proof of duplication.
Multiple entries	Registration	Describe login/registration controls for closed surveys.	Section 2.2 and S4 Section S20: Google sign-in was not required and the survey was open.
Analysis	Handling of incomplete questionnaires	Describe inclusion/exclusion of incomplete or early-terminated questionnaires.	Sections 2.2, 2.5 and 3.1: one non-consenting and 15 eligibility-gate records had no subsequent questionnaire data and were excluded. Required responses were complete. Literal None certification responses were restored from the original export after a processing error; no additional participants were excluded and no imputation was used.
Analysis	Atypical timestamp	State whether submissions were excluded by completion time/timestamp.	No completion-time exclusion criterion was used. Section 2.5 reports only the author’s manual age–experience and age–highest-qualification checks; no participant was excluded on this basis. Identical-pattern and low-engagement screening were not performed. Exact timestamps are absent from the shared dataset.
Analysis	Statistical correction	Describe weighting/propensity correction for non-representative sampling, if used.	Sections 2.2 and 4.6; S4 Section S20: no survey weighting or propensity adjustment. Convenience sampling limits generalizability; pooled descriptive results are provided in S4 Table S18a.

Checklist source: Eysenbach G. Improving the Quality of Web Surveys: The Checklist for Reporting Results of Internet E-Surveys (CHERRIES). J Med Internet Res. 2004;6(3):e34. doi:10.2196/jmir.6.3.e34.
